# Diagnostic significance of HRCT imaging features in adult mycoplasma pneumonia: a retrospective study

**DOI:** 10.1038/s41598-023-50702-3

**Published:** 2024-01-02

**Authors:** Dong-xin Sui, Han-chen Ma, Chao-chao Wang, Hong-yan Shao, Shao-hua Xu, Ning-ning Fang

**Affiliations:** 1 Department of Respiration, The Second Hospital of Shandong University, Jinan, China; 2 Department of Anesthesiology, Qilu Hospital of Shandong University, No. 107, Wenhua Xi Road, Jinan, 250012 Shandong China

**Keywords:** Infection, Infectious diseases

## Abstract

Mycoplasma pneumoniae pneumonia (MPP) often overlaps with the clinical manifestations and chest imaging manifestations of other types of community-acquired pneumonia (CAP). We retrospectively analyzed the clinical and imaging data of a group of patients with CAP, summarized their clinical and imaging characteristics, and discussed the diagnostic significance of their certain HRCT findings. The HRCT findings of CAP researched in our study included tree-in-bud sign (TIB), ground-glass opacity (GGO), tree fog sign (TIB + GGO), bronchial wall thickening, air-bronchogram, pleural effusion and cavity. The HRCT findings of all cases were analyzed. Among the 200 cases of MPP, 174 cases showed the TIB, 193 showed the GGO, 175 showed the tree fog sign, 181 lacked air-bronchogram. In case taking the tree fog sign and lack of air-bronchogram simultaneously as an index to distinguish MPP from OCAP, the sensitivity was 87.5%, the specificity was 97.5%, the accuracy was 92.5%. This study showed that that specific HRCT findings could be used to distinguish MPP from OCAP. The combined HRCT findings including the tree fog sign and lacked air-bronchogram simultaneously would contribute to a more accurate diagnosis of MPP.

## Introduction

Mycoplasma pneumoniae pneumonia (MPP) is a respiratory tract infectious disease caused by Mycoplasma pneumoniae. Mycoplasma pneumoniae was a common cause of respiratory tract infections before the COVID-19 pandemic, with worldwide incidence of 8.61% from 2017 to 2020, measured by direct test methods. However, non-pharmaceutical interventions (NPIs) against COVID-19 drastically lowered the transmission of M pneumoniae. In the short term, the incidence was down to 1.69% world-widely between 2020 and 2021, but the specific epidemiology varies by region^1^. Mycoplasma pneumoniae is now still considered a common cause of community-acquired pneumonia and estimates show that around 1% of the population of the United States is infected annually^2^. In China, Streptococcus pneumoniae, Mycoplasma pneumoniae and Klebsiella pneumoniae are the three leading bacterial pathogens (29.9%, 18.6% and 15.8%) of acute respiratory infections^3^. Because MP infection often overlaps with the clinical manifestations and chest imaging manifestations of other types of CAP, an early diagnosis or exclusion of MPP can result in more accurate treatment for CAP patients. However, mycoplasma pneumoniae cannot be detected by Gram staining or routine bacterial culture. Moreover, there is currently no rapid serological test with sufficient performance to detect mycoplasma pneumoniae, and a rapid antigen test has demonstrated poor sensitivity^4,5^. Therefore, the diagnosis of MPP requires elevated serum antibody levels, and therefore, it can take 2 or more weeks to obtain definitive results. MPP is an acute lung infection mainly with interstitial lesions. Due to the significant differences in clinical manifestations between MPP and pneumonia caused by common bacteria such as Streptococcus pneumoniae, and the treatment with antibiotics such as β-lactams and sulfonamide drugs is ineffective, early identification of MPP is crucial for the initial treatment of CAP^6–10^. For the early diagnosis of MPP, medical imaging is a powerful supplement to etiological examination. In earlier study, medical imaging has been used to determine the etiology of pneumonia^11^. Some studies using chest roentgenogram or computed tomography (CT) described central zone predominance of abnormalities observed in MPP^12,13^. Characteristic high-resolution CT (HRCT) findings in MPP were also reported^14,15^. However, only a few reports, with a relatively small number of patients, described the differences in HRCT findings between MPP and other CAP (OCAPs) ^16,17^. This study retrospectively analyzed the clinical and imaging data of a group of patients with CAP, summarized their clinical and imaging characteristics, and discussed the diagnostic significance of their certain HRCT findings, including "Tree-in-Bud Sign(TIB)", "Ground-glass opacity (GGO)", "Tree Fog Sign (TIB + GGO)", "Bronchial wall thickening", "Air-bronchogram", “Pleural effusion" and "Cavity".

## Materials and methods

### Materials

We retrospectively enrolled 400 patients who had community acquired pneumonia (CAP) with a confirmed etiology and underwent HRCT between January 2021 and January 2022. As mentioned in recent similar studies^12–18^, it was estimated that the incidence of certain imaging features such as “Bronchial wall thickening”, “Intralobular or lobular GGO” in MPP exceeded 70–90%, while the incidence in OCAP was 35–60%. According to these studies, using PASS 15 software, we calculated the sample size based on a 1:1 ratio for two groups, with a sample size of approximately 200 cases per group. Therefore, our study selected 200 clinically confirmed cases of MPP and 200 cases of OCAP as a control. The study was approved by the Ethics Committee of the second hospital of Shandong university (Approval number: KYLL-2022LW132, provided in the supplementary file). All procedures were carried out in accordance with the principles of the Helsinki Declaration. The informed consent was obtained from all subjects and/or their legal guardians.

Inclusion criteria:

All cases met the diagnostic criteria of community-acquired pneumonia: A. Onset in community. B. Relevant clinical manifestations of pneumonia: (1) New onset of cough or expectoration, or aggravation of existing symptoms of respiratory tract diseases, with or without purulent sputum, chest pain, dyspnea, or hemoptysis; (2) Fever; (3) Signs of pulmonary consolidation and/or moist rales; (4) Peripheral white blood cell count (WBC) > 10 × 10^9^/L or < 4 × 10^9^/L, with or without a left shift. C. Chest radiograph showing new patchy infiltrates, lobar or segmental consolidation, ground-glass opacities, or interstitial changes, with or without pleural effusion. Clinical diagnosis can be established if a patient satisfies Criterion A, Criterion C and any one condition of Criterion B and meanwhile, tuberculosis, pulmonary tumour, noninfectious interstitial lung disease, pulmonary edema, atelectasis, pulmonary embolism, pulmonary eosinophilia and pulmonary vasculitis are all excluded^19,20^. The HRCT image was the initial image scanned by the patient at the first visit without treatment. Sputum examination, urinary antigen testing for Streptococcus pneumoniae, and serology for Mycoplasma pneumoniae were performed. A blood culture was also performed for suspected bacteremia. Sputum was obtained by spontaneous expectoration or with the aid of nebulized saline and was examined by Gram and Ziehl–Neelsen staining and used to culture for bacteria and acid-fast bacilli. There are many laboratory testing methods for mycoplasma pneumoniae. During recent years, various new techniques have been adapted for the diagnosis of mycoplasma pneumoniae infection, notably in the field of molecular biology. Standard polymerase chain reaction (PCR) is currently the method of choice for direct pathogen detection. Serology, which is the basic strategy for mycoplasma diagnosis in routine clinical practice, has been improved by the widespread availability of sensitive assays for separate detection of different antibody classes. For the diagnosis of mycoplasma pneumonia, serology and direct pathogen detection should be combined^21–23^. In this study, according to currently universally acknowledged standards, those who met the diagnostic criteria for CAP and serologically positive were diagnosed with MPP. Serologically positive identification of mycoplasma pneumoniae based on a positive immunoglobulin M (IgM) result or a fourfold increase in immunoglobulin G (IgG) levels in convalescent versus initial blood samples by chemiluminescence immunoassay^21–23^. Other etiologies including Coxiella burnetii, Legionella pneumophila, Chlamydophila psittaci, and Chlamydophila pneumoniae were examined, as deemed necessary by the attending physicians.

Exclusion criteria:

A. Poor CT image quality. B. Patients who had an unclear etiology. C. Patients with underlying pulmonary diseases, aspiration pneumonia, malignant diseases, connective tissue diseases or immunocompromised status were also excluded.

### Methods

#### Study design

The clinical characteristics and chest HRCT images of 400 patients were analyzed retrospectively. The clinical characteristics included gender, age, height, weight, body mass index (BMI), smoking history, maximum body temperature and one or more accompanying systemic symptoms (headache, muscle soreness, fatigue, anorexia, etc.). The HRCT findings of CAP researched in our study included tree-in-bud sign (TIB), ground-glass opacity (GGO), tree fog sign (TIB + GGO), bronchial wall thickening, air-bronchogram, pleural effusion and cavity.

#### Radiologic data

We have provided partial raw data in the supplementary file, and some certain data cannot be provided to protect the patients’ privacy. The datasets used and/or analysed during the current study available from the corresponding author on reasonable request. The image data of this study were read by two senior radiologists and one chief physician of respiratory medicine. The HRCT findings of all cases were analyzed. In case of disagreement or dispute, a third senior radiologist was asked for consultation and discussion.

#### Statistical analysis

The enumeration data were expressed by frequency or rate (%), and chi-square test was used for comparison between groups. The measurement data were expressed by means ± standard deviation (x ± sd), and ANOVA (analysis of variance) was used for comparison between groups. All tests were two-tailed and a *P* value < 0.05 was considered significant.

### Ethics approval

The study was approved by the Ethics Committee of the second hospital of Shandong university (No. KYLL-2022LW132, provided in the supplementary file). All procedures were carried out in accordance with the principles of the Helsinki Declaration. The informed consent was obtained from all subjects and/or their legal guardians.

## Results

### Enrolled patients

Clinical parameters are presented in Table 1. In this study, none patient was complicated with other basic diseases. Most patients came to the hospital due to cough, expectoration and fever, without dyspnea or respiratory failure. The differences in clinical characteristics, including gender, age, height, weight, body mass index (BMI), smoking history, maximum body temperature and accompanying systemic symptoms (headache, muscle soreness, fatigue, anorexia, etc.), were not statistically significant between MPP group and OCAP group (*P* > 0.05).

**Table 1:** Clinical parameters of included cases. Data are presented as mean ± SD for continuous variables and as numbers or proportions for categorical variables.

	MMP (n = 200)	OCAP (n = 200)	F/χ^2^	*P* value
Male	100	135	2.527	0.112
Age, years (mean ± SD)	45.98 ± 18.57	42.8 ± 19.45	2.877	0.091
BMI (mean ± SD)	25.95 ± 2.78	26.17 ± 2.65	0.663	0.416
Body weight (mean ± SD)	71.55 ± 10.59	72.83 ± 10.46	1.468	0.226
Height (mean ± SD)	165.75 ± 6.97	166.53 ± 7.56	1.136	0.287
Temperature (mean ± SD)	38.47 ± 0.87	38.48 ± 0.84	0.014	0.907
Smoking history	75	85	0.208	0.648
Systemic symptoms	80	80	0	1

### Results of the analysis of HRCT findings

#### Tree-in-bud sign (TIB)

**Figure 1:**
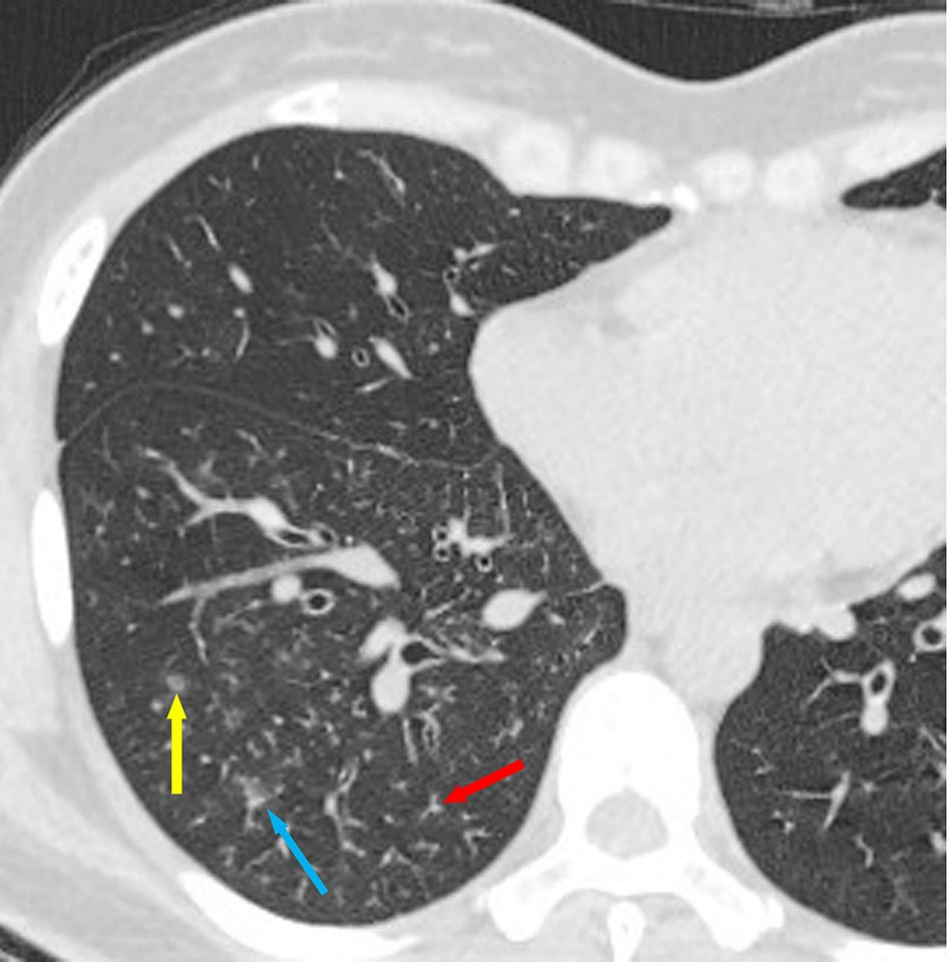
HRCT findings of a 30-year-old female case with mycoplasma pneumoniae pneumonia. The red arrow showed the tree-in-bud sign (TIB). The yellow arrow showed the ground-glass opacity (GGO). The blue arrow showed the tree fog sign (TIB + GGO).

Among the 200 cases of MPP, 174 cases showed the tree-in-bud sign (TIB) (Fig. 1, red arrow), accounting for 87%, compared with 62% in OCAP (124/200). The tree-in-bud sign (TIB) was often observed in MPP patients, and was also observed in some bronchiolitis patients. In case taking the tree-in-bud sign (TIB) as an index to distinguish MPP from OCAP, the sensitivity was 88%, the specificity was 38%, the accuracy was 63%, the odds ratio was 4.2, and chi-square test *p* = 0.01 (Table 2).

**Table 2:** Comparison of HRCT findings between two groups.

HRCT findings	MMP (n = 200)	OCAP (n = 200)	Sensitivity	Specificity	Accuracy	OR	*P* value (Chi square test)
Tree-in-bud sign (TIB)	174	124	0.88	0.38	0.63	4.20	0.01
GGO	193	101	0.98	0.50	0.74	39.00	< 0.001
TIB + GGO (tree frog sign)	174	76	0.88	0.63	0.75	11.67	< 0.001
Bronchial wall thickening	146	151	0.73	0.25	0.49	0.88	0.799
Less air-bronchogram	181	80	0.93	0.60	0.76	18.50	< 0.001
Pleural involvement	123	150	0.60	0.25	0.43	0.50	0.152
Less pleural effusion	195	177	0.98	0.13	0.55	5.57	0.09
Single lobe	110	123	0.55	0.38	0.46	0.73	0.496
Multiple lobes	91	76	0.45	0.63	0.54	1.36	0.496
Less cavity	197	125	0.98	0.38	0.68	23.40	0.873

#### Ground-glass opacity (GGO)

Among the 200 cases of MPP, 193 cases showed the ground-glass opacity (GGO) (Fig. 1, yellow arrow), accounting for 96.5%, compared with 50.5% in OCAP (101/200). The ground-glass opacity (GGO) was often observed in MPP patients, and was also observed in some OBP patients. In case taking the ground-glass opacity (GGO) as an index to distinguish MPP from OCAP, the sensitivity was 98%, the specificity was 50%, the accuracy was 74%, the odds ratio was 39, and chi-square test *p* < 0.001 (Table 2).

#### Tree fog sign (TIB + GGO)

The tree fog sign, which means that both tree-in-bud sign and ground-glass opacity exist simultaneously, indicates that the edges of the tree-in-bud are not clear and there is exudative change. Only when the GGO surrounds the TIB can be determined "tree fog sign", and when they are simultaneously displayed but separately distributed, they could not be determined "tree fog sign". Among the 200 cases of MPP, 174 cases showed the tree fog sign (TIB + GGO) (Fig. 1, blue arrow), accounting for 87%, compared with 38% in OCAP (76/200). The tree fog sign (TIB + GGO) was often observed in MPP patients, and was also observed in some OBP patients. In case taking the tree fog sign (TIB + GGO) as an index to distinguish MPP from OCAP, the sensitivity was 88%, the specificity was 63%, the accuracy was 75%, the odds ratio was 11.67, and chi-square test *p* < 0.001 (Table 2).

#### Air-bronchogram

**Figure 2:**
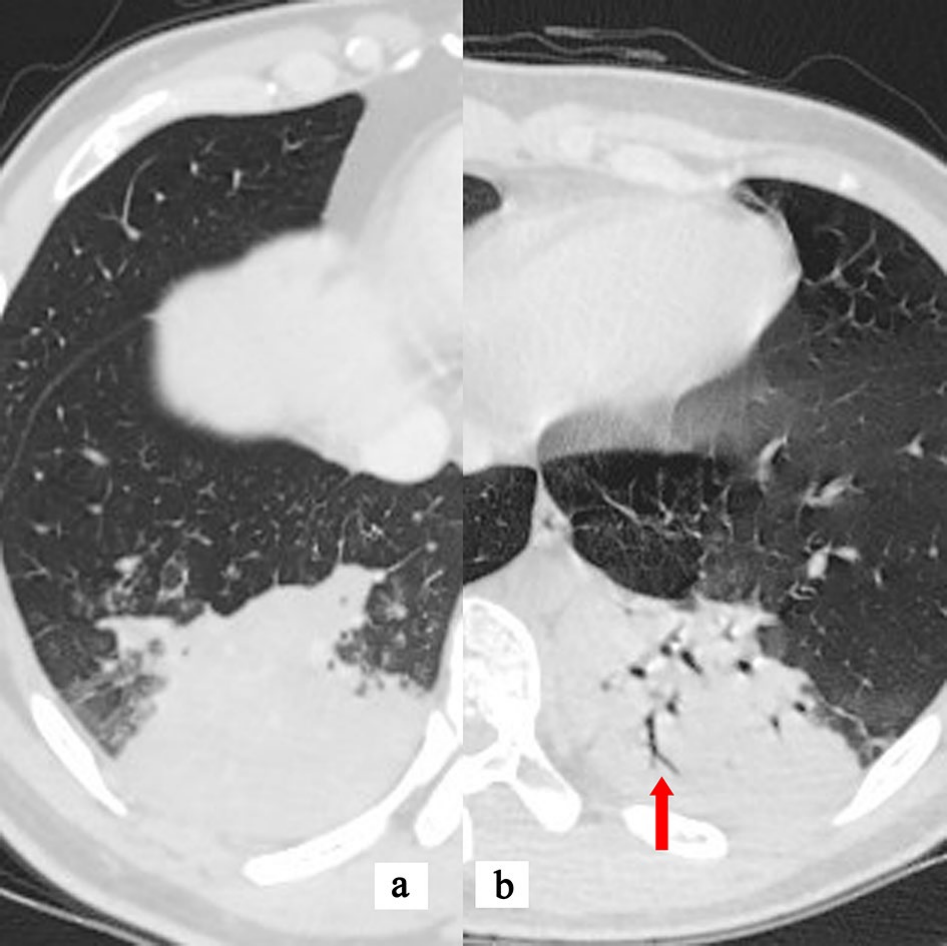
**(a)** HRCT findings of a 38-year-old male case with mycoplasma pneumoniae pneumonia. **(b)** HRCT findings of a 33-year-old male case with pneumococcal pneumonia. The red arrow showed the air-bronchogram.

Among the 200 cases of MPP, 181 lacked air-bronchogram (Fig. 2, the red arrow showed the air-bronchogram), accounting for 90.5%, compared with 40% in OCAP (80/200). Air-bronchogram was often observed in some OCAP patients (Fig. 2B, the red arrow showed the air-bronchogram) but was rarely observed in MPP patients (Fig. 2A). In case taking lack of air-bronchogram as an index to distinguish MPP from OCAP, the sensitivity was 93%, the specificity was 60%, the accuracy was 76%, the odds ratio was 18.5, and chi-square test *p* < 0.001 (Table 2).

#### Tree fog sign (TIB + GGO) + lack of air-bronchogram

Among the 200 cases of mycoplasma pneumonia, 175 cases had the tree fog sign and lacked air-bronchogram simultaneously, accounting for 87.5%, compared with 2.5% in OCAP (5/200). In case taking the tree fog sign (TIB + GGO) and lack of air-bronchogram simultaneously as an index to distinguish MPP from OCAP, the sensitivity was 87.5%, the specificity was 97.5%, the accuracy was 92.5%, the odds ratio was 273, and chi-square test *p* < 0.001 (Table 3).

**Table 3:** In case taking the tree fog sign (TIB + GGO) and lack of air-bronchogram simultaneously as an index to distinguish MPP from OCAP, the sensitivity was 87.5%, the specificity was 97.5%, the accuracy was 92.5%, the odds ratio was 273, and chi-square test *p* < 0.001.

HRCT findings	MMP (n = 200)	OCAP (n = 200)	Sensitivity	Specificity	Accuracy	OR	*P* value (Chi square test)
TIB + GGO (Tree Frog Sign) + Less air-bronchogram	175	5	0.875	0.975	0.925	273.00	< 0.001

#### Other HRCT findings

**Figure 3:**
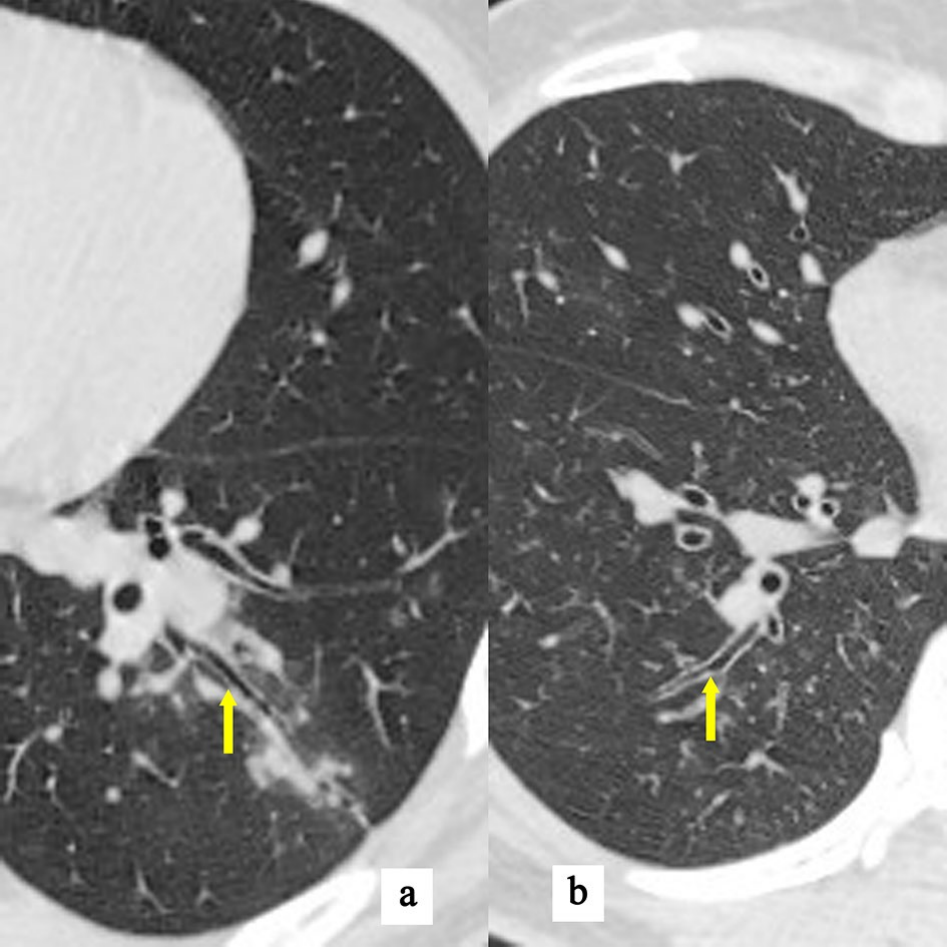
HRCT findings of a 41-year-old male case with mycoplasma pneumoniae pneumonia. The yellow arrow showed the bronchial wall thickening.

**Figure 4:**
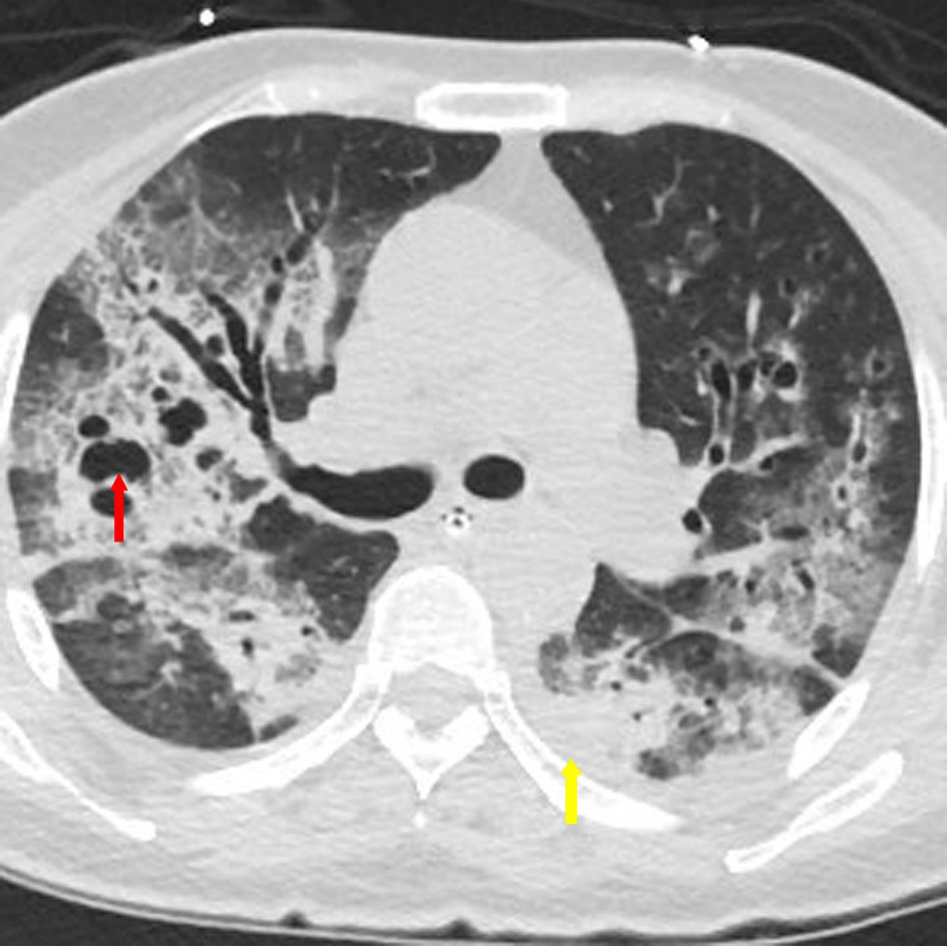
HRCT findings of a 65-year-old female case with staphylococcus aureus pneumonia. The red arrow showed the cavity, the yellow arrow showed the pleural effusion.

It has been reported that the bronchial wall thickening was a more specific sign of MPP^23^ while our study found that the specificity was weak (Fig. 3, yellow arrow). The other HRCT findings, including the distribution of lesions, pleural effusion and cavity (Fig. 4, the red arrow showed the cavity, the yellow arrow showed the pleural effusion), were not statistically significant between MPP group and OCAP group (*p* > 0.05) (Table 2).

## Discussion

This study showed that that specific HRCT findings could be used to distinguish MPP from OCAP. The tree-in-bud sign (TIB), the ground-glass opacity (GGO), the tree fog sign (TIB + GGO) and the lack of air-bronchogram were significant findings in MPP.

As previously reported, bronchial wall thickening was characteristic findings of MPP on HRCT^14,15^ while our study found that the specificity was weak. As known, the cause of bronchial wall thickening is bronchial inflammation. We determined that a considerable number of OCAPs might also invade the bronchus and cause bronchitis or bronchiolitis which induced the bronchial wall thickening as a HRCT finding.

Centrilobular nodules on CT correspond pathologically to cellular infiltration in bronchioles with exudates or granulation tissue in bronchiolar lumens. This HRCT finding was generally named the tree-in-bud sign (TIB). A previous study showed the microbiological features of CAP patients with a TIB pattern share similarities with those of pneumonia in the general population^24^. In another study, Mycoplasma pneumoniae infection was frequently associated with a TIB pattern^25^. Mycoplasma pneumoniae has an affinity for airway cilia and bronchioles and causes peribronchial and perivascular infiltration of mononuclear cells and edematous and ulcerative lesions in bronchial walls^26^. In the present study, 87% of MPP cases had TIB pattern, compared with 62% in OCAP. The ground-glass opacity (GGO) indicates inflammatory exudation. Among the 200 cases of MPP, 193 cases showed the ground-glass opacity (GGO), accounting for 96.5%, compared with 50.5% in OCAP. In this study, the HRCT findings of GGO combined with TIB, defined as the tree fog sign, appeared in 174 MPP cases, accounting for 87%, compared with 38% in OCAP (76/200). The above results showed that the tree-in-bud sign (TIB), the ground-glass opacity (GGO) and the tree fog sign (TIB + GGO) were relatively specific HRCT characters of MPPs, but still part of OCAPs also had the above findings.

It is well known that Mycoplasma pneumoniae infection often involves bronchioles. HRCT findings of diffusely narrowed air bronchograms and volume loss of the involved lobe have been reported in MPP^14^. The bronchial lumen infected by Mycoplasma pneumoniae could be narrowed and obstructed by the thickened bronchial wall and mucus production. Therefore, in MPP, the air-bronchogram on HRCT could be narrowed or absent and lead to volume loss in the infiltrated area due to decreased air or subsequent atelectasis. In our study, among the 200 cases of MPP, 181 lacked air-bronchogram, accounting for 90.5%, compared with 40% in OCAP (80/200). Air-bronchogram was often observed in some OCAP patients but was rarely observed in MPP patients. Therefore, the lack of air-bronchogram was a significant finding in MPP. However, this pathologic change can also occur during other infections^27^. With many other causative bacteria of pneumonia, such as streptococcus pneumoniae, the areas primarily affected are the alveoli. Radiologically, air space consolidation by streptococcus pneumoniae typically abuts the surrounding visceral pleura and is likely to form air bronchograms in consolidation^28^. Additionally, a few studies have reported that such findings in MPP are attributable to organizing pneumonia^29^.

In our study, the differences in clinical characteristics, including gender, age, height, weight, body mass index (BMI), smoking history, maximum body temperature and accompanying systemic symptoms (headache, muscle soreness, fatigue, anorexia, etc.), were not statistically significant between MPP group and OCAP group. Therefore, it is not accurate to distinguish MPP from OCAP only by clinical characteristics. The detection of pathogenic microorganisms is the gold standard for the diagnosis of MPP. Identification of Mycoplasma pneumoniae based on a positive immunoglobulin M (IgM) result or a fourfold increase in immunoglobulin G (IgG) levels in convalescent versus initial blood samples by chemiluminescence immunoassay or positivity for mycoplasma pneumoniae in a sputum by polymerase chain reaction (PCR)^23^. HRCT is not considered the gold standard for diagnosing MPP. It may be difficult to distinguish MPP from OBP completely using HRCT, and the interpretations of HRCT findings lack the objectivity of a quantitative analysis. However, HRCT is an important supplementary method for the diagnosis of MPP. We researched some relatively specific HRCT findings of MPP, including tree-in-bud sign (TIB), ground-glass opacity (GGO), tree fog sign (TIB + GGO), bronchial wall thickening, air-bronchogram, pleural effusion and cavity. Similar results could be seen from the study by Nakanishi et al., which considered that HRCT had considerable ability to detect a lateral bronchial abnormality and to diagnose or rule out MPP based on the distribution of affected areas, abnormalities in lateral bronchial lesions, and the degree of air bronchogram in the infiltrates^18^. However, the diagnosis of MPP by these methods was not simple^30^. We therefore considered that the combined HRCT findings would contribute to a more accurate diagnosis of MPP. That was, community-acquired pneumonia (CAP) with the following HRCT features including the tree fog sign (TIB + GGO) and lacked air-bronchogram simultaneously indicated mycoplasma pneumoniae infection closely.

There were several limitations in this study. Firstly, our study was a single-center study. Secondly, the number of cases involved were limited. Thirdly, current diagnostic PCR or serology cannot discriminate between mycoplasma pneumoniae infection and carriage. Therefore, the diagnostic criteria for MPP needs further optimization.

## Conclusions

This study showed that specific HRCT findings could be used to distinguish MPP from OCAP. The combined HRCT findings including the tree fog sign (TIB + GGO) and lacked air-bronchogram simultaneously would contribute to a more accurate diagnosis of MPP.

### Supplementary Information

Supplementary Information 1.

Supplementary Information 2.

## Data Availability

We have provided partial raw data in the supplementary file, and some certain data cannot be provided to protect the patients’ privacy. The datasets used and/or analysed during the current study available from the corresponding author on reasonable request.
