# Supplementary figures and images for: Diagnostic significance of HRCT imaging features in adult mycoplasma pneumonia: a retrospective study (part 2 of 2)
Source: Sci Rep. 2024 Jan 2;14:153. doi: 10.1038/s41598-023-50702-3 (PMC10761950; doi:10.1038/s41598-023-50702-3)

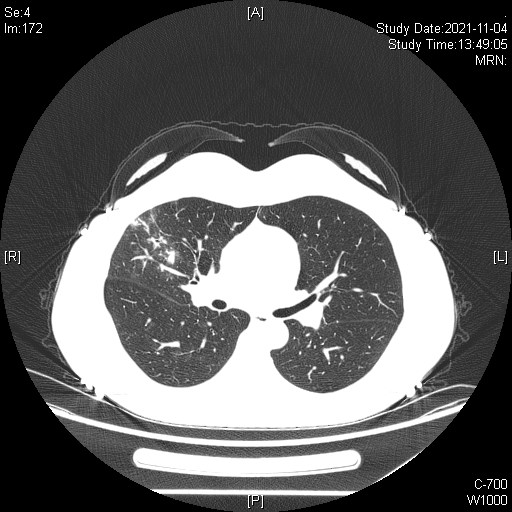

Supplement: Supplementary file 2 — Supplementary Information 2. [file 41598_2023_50702_MOESM2_ESM.zip › image data/MPP/zhang2/zhang (7).jpg]

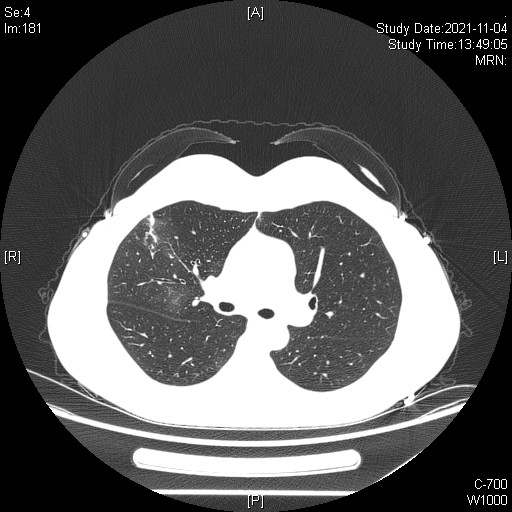

Supplement: Supplementary file 2 — Supplementary Information 2. [file 41598_2023_50702_MOESM2_ESM.zip › image data/MPP/zhang2/zhang (8).jpg]

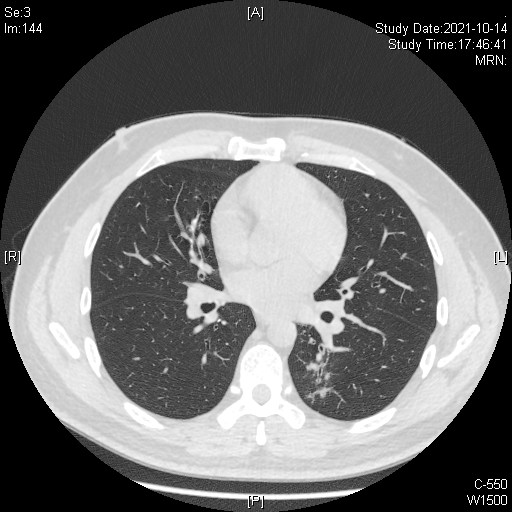

Supplement: Supplementary file 2 — Supplementary Information 2. [file 41598_2023_50702_MOESM2_ESM.zip › image data/MPP/zhao/zhao (1).jpg]

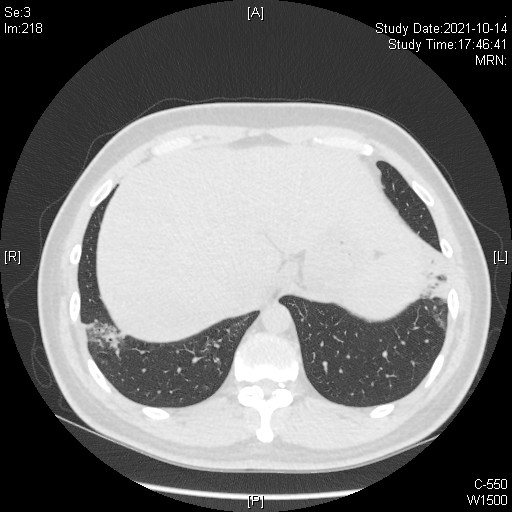

Supplement: Supplementary file 2 — Supplementary Information 2. [file 41598_2023_50702_MOESM2_ESM.zip › image data/MPP/zhao/zhao (10).jpg]

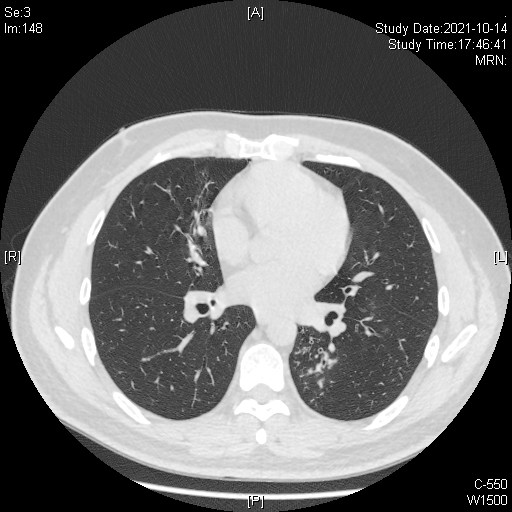

Supplement: Supplementary file 2 — Supplementary Information 2. [file 41598_2023_50702_MOESM2_ESM.zip › image data/MPP/zhao/zhao (2).jpg]

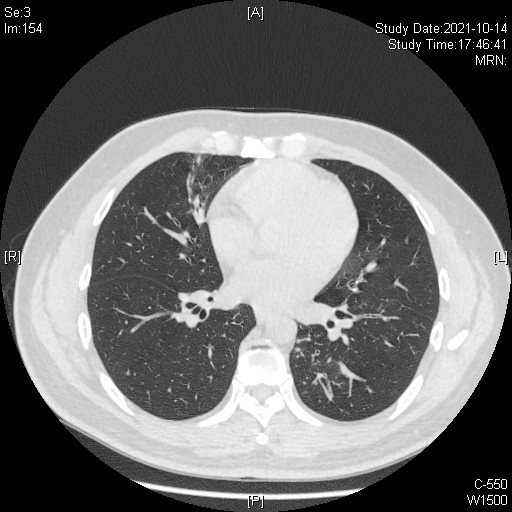

Supplement: Supplementary file 2 — Supplementary Information 2. [file 41598_2023_50702_MOESM2_ESM.zip › image data/MPP/zhao/zhao (3).jpg]

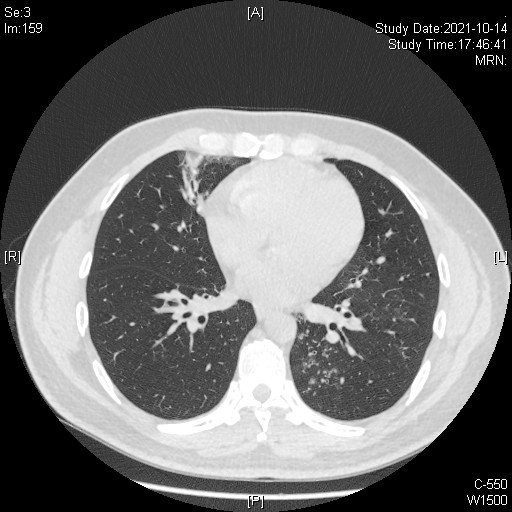

Supplement: Supplementary file 2 — Supplementary Information 2. [file 41598_2023_50702_MOESM2_ESM.zip › image data/MPP/zhao/zhao (4).jpg]

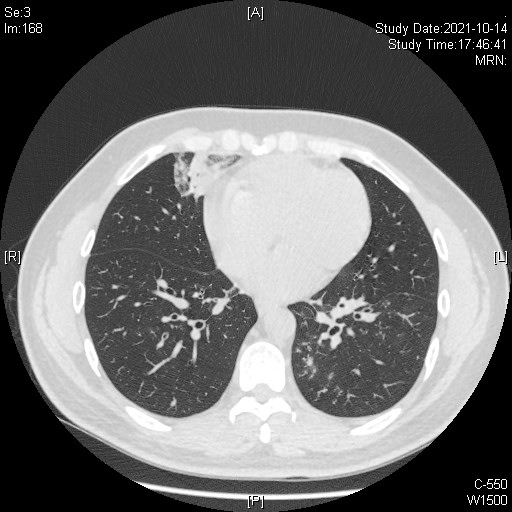

Supplement: Supplementary file 2 — Supplementary Information 2. [file 41598_2023_50702_MOESM2_ESM.zip › image data/MPP/zhao/zhao (5).jpg]

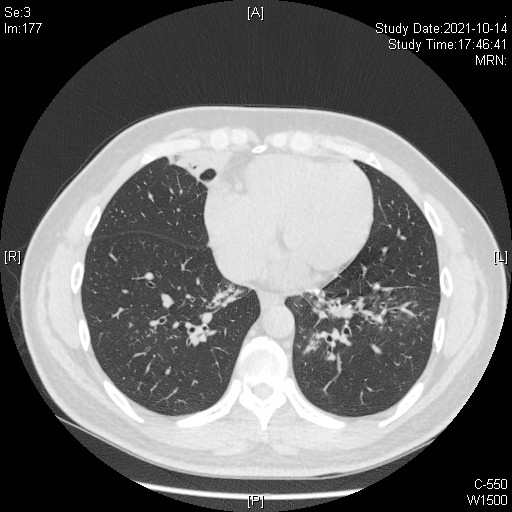

Supplement: Supplementary file 2 — Supplementary Information 2. [file 41598_2023_50702_MOESM2_ESM.zip › image data/MPP/zhao/zhao (6).jpg]

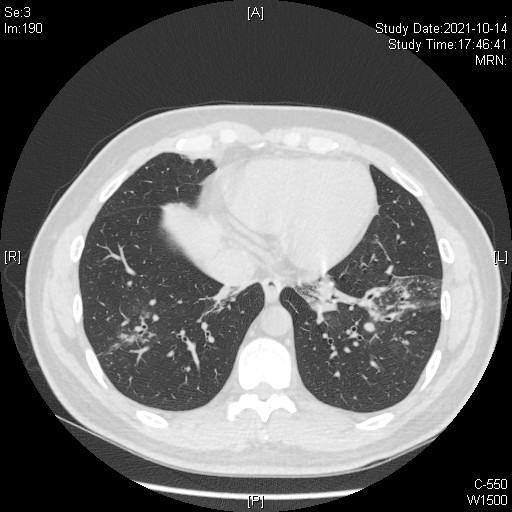

Supplement: Supplementary file 2 — Supplementary Information 2. [file 41598_2023_50702_MOESM2_ESM.zip › image data/MPP/zhao/zhao (7).jpg]

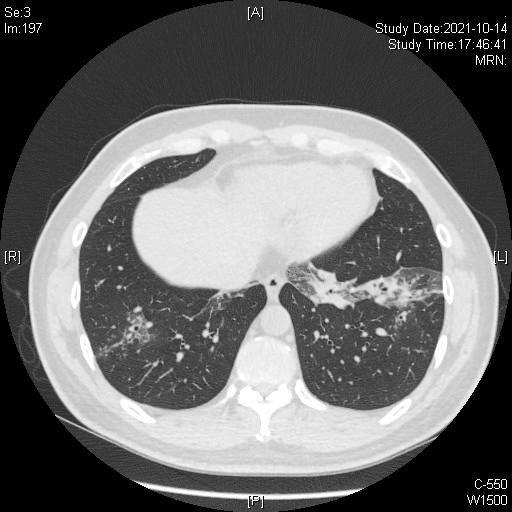

Supplement: Supplementary file 2 — Supplementary Information 2. [file 41598_2023_50702_MOESM2_ESM.zip › image data/MPP/zhao/zhao (8).jpg]

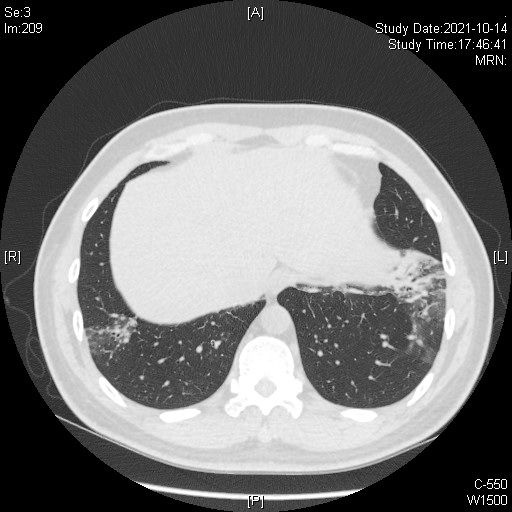

Supplement: Supplementary file 2 — Supplementary Information 2. [file 41598_2023_50702_MOESM2_ESM.zip › image data/MPP/zhao/zhao (9).jpg]

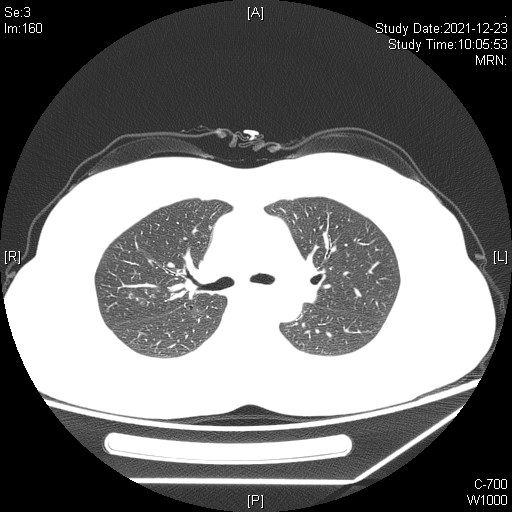

Supplement: Supplementary file 2 — Supplementary Information 2. [file 41598_2023_50702_MOESM2_ESM.zip › image data/MPP/zhao2/zhao (1).jpg]

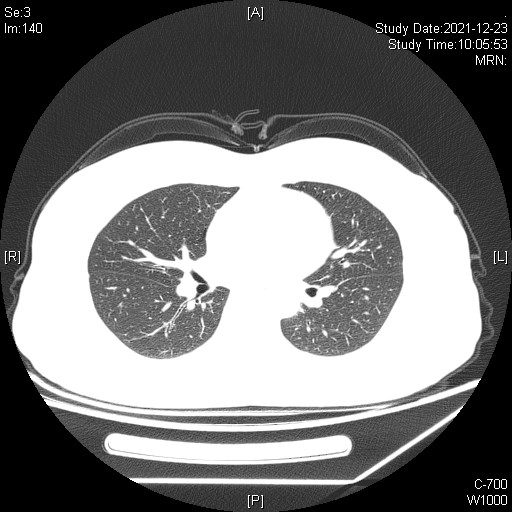

Supplement: Supplementary file 2 — Supplementary Information 2. [file 41598_2023_50702_MOESM2_ESM.zip › image data/MPP/zhao2/zhao (2).jpg]

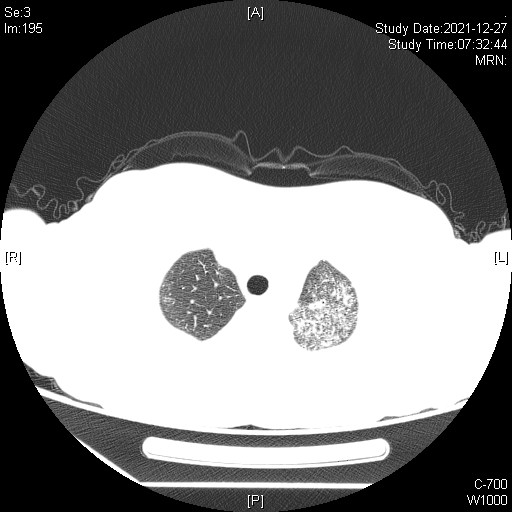

Supplement: Supplementary file 2 — Supplementary Information 2. [file 41598_2023_50702_MOESM2_ESM.zip › image data/OCAP/cui/cui (1).jpg]

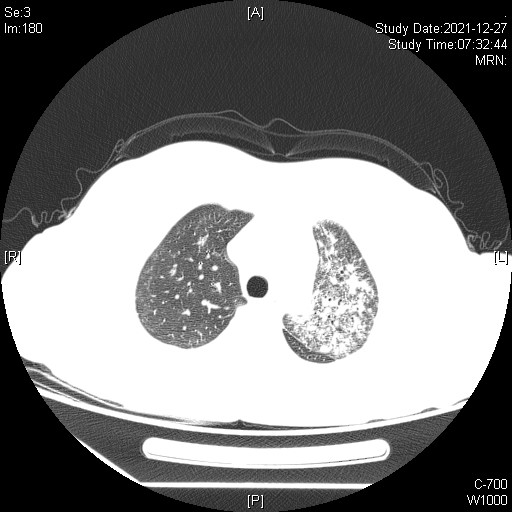

Supplement: Supplementary file 2 — Supplementary Information 2. [file 41598_2023_50702_MOESM2_ESM.zip › image data/OCAP/cui/cui (2).jpg]

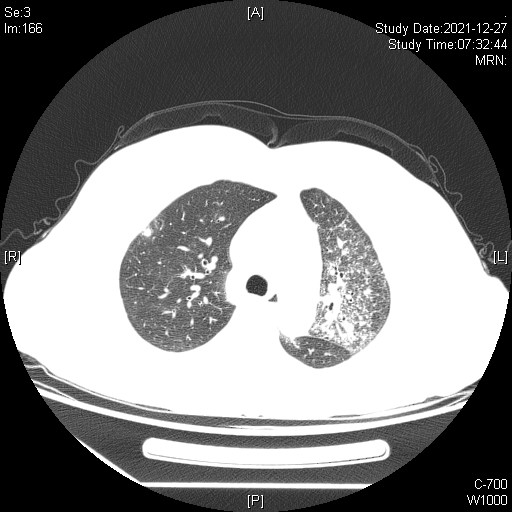

Supplement: Supplementary file 2 — Supplementary Information 2. [file 41598_2023_50702_MOESM2_ESM.zip › image data/OCAP/cui/cui (3).jpg]

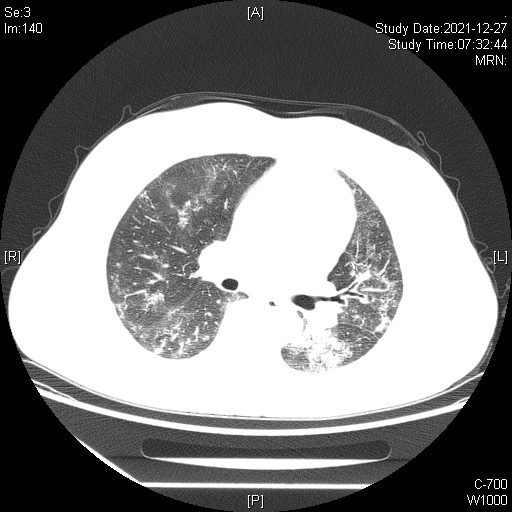

Supplement: Supplementary file 2 — Supplementary Information 2. [file 41598_2023_50702_MOESM2_ESM.zip › image data/OCAP/cui/cui (4).jpg]

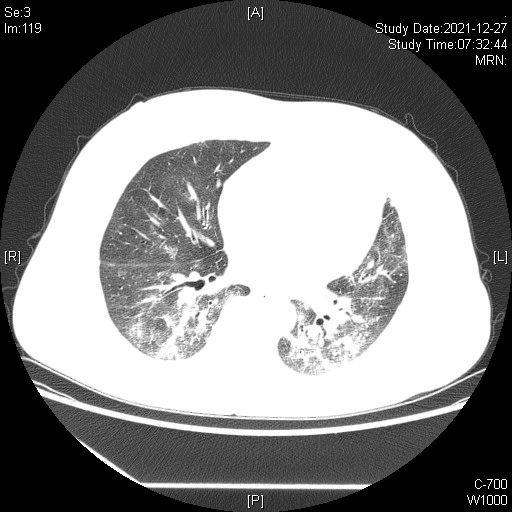

Supplement: Supplementary file 2 — Supplementary Information 2. [file 41598_2023_50702_MOESM2_ESM.zip › image data/OCAP/cui/cui (5).jpg]

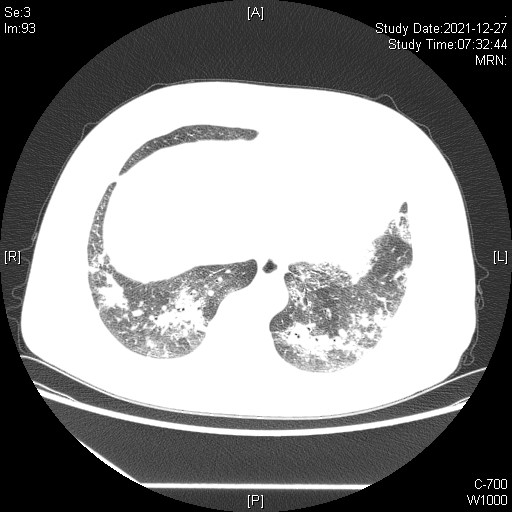

Supplement: Supplementary file 2 — Supplementary Information 2. [file 41598_2023_50702_MOESM2_ESM.zip › image data/OCAP/cui/cui (6).jpg]

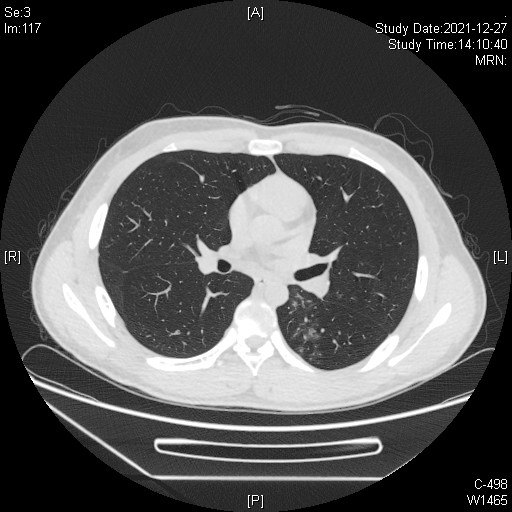

Supplement: Supplementary file 2 — Supplementary Information 2. [file 41598_2023_50702_MOESM2_ESM.zip › image data/OCAP/du/du (1).jpg]

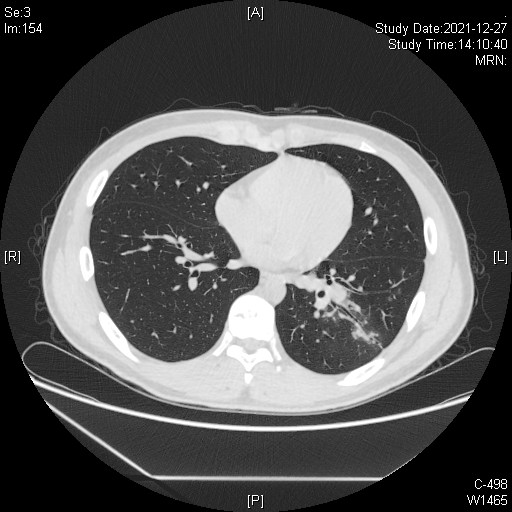

Supplement: Supplementary file 2 — Supplementary Information 2. [file 41598_2023_50702_MOESM2_ESM.zip › image data/OCAP/du/du (2).jpg]

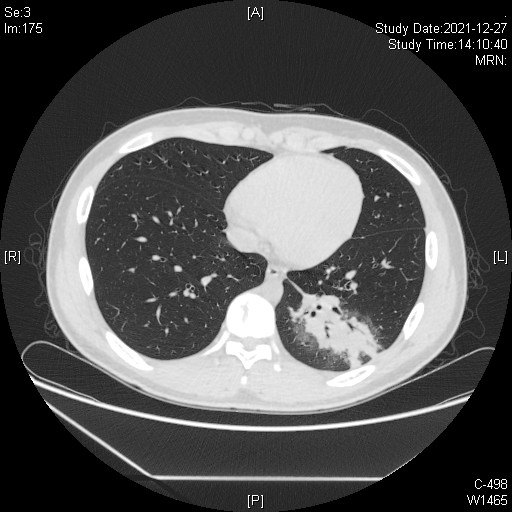

Supplement: Supplementary file 2 — Supplementary Information 2. [file 41598_2023_50702_MOESM2_ESM.zip › image data/OCAP/du/du (3).jpg]

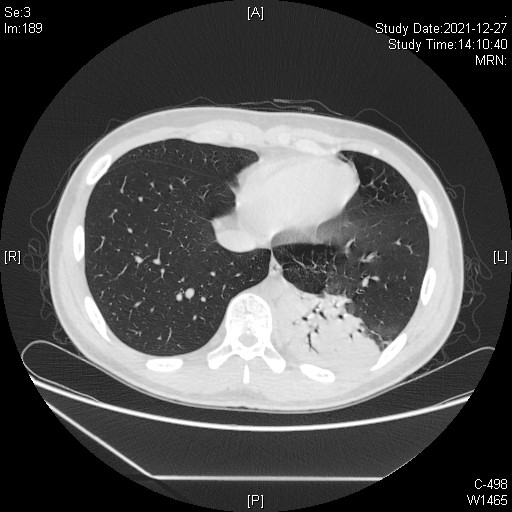

Supplement: Supplementary file 2 — Supplementary Information 2. [file 41598_2023_50702_MOESM2_ESM.zip › image data/OCAP/du/du (4).jpg]

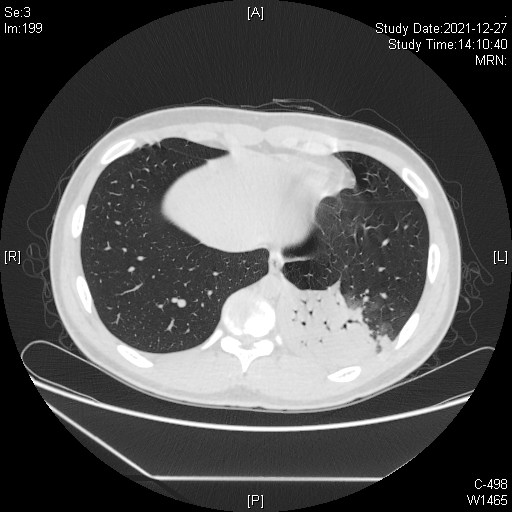

Supplement: Supplementary file 2 — Supplementary Information 2. [file 41598_2023_50702_MOESM2_ESM.zip › image data/OCAP/du/du (5).jpg]

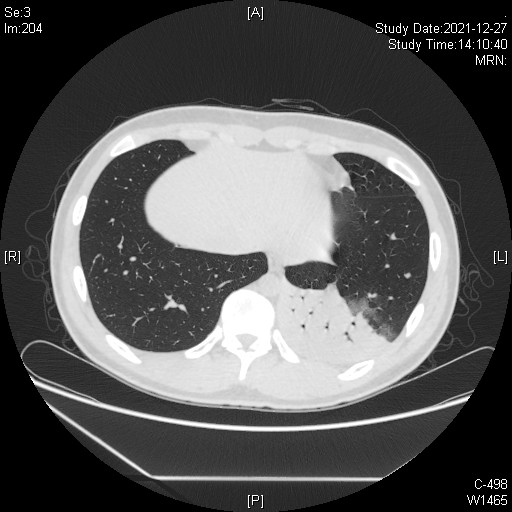

Supplement: Supplementary file 2 — Supplementary Information 2. [file 41598_2023_50702_MOESM2_ESM.zip › image data/OCAP/du/du (6).jpg]

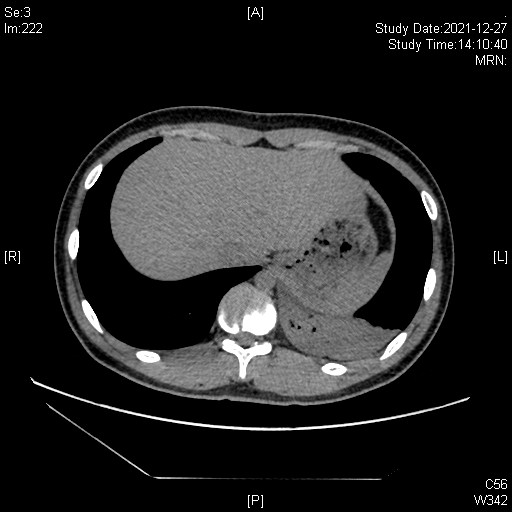

Supplement: Supplementary file 2 — Supplementary Information 2. [file 41598_2023_50702_MOESM2_ESM.zip › image data/OCAP/du/du (7).jpg]

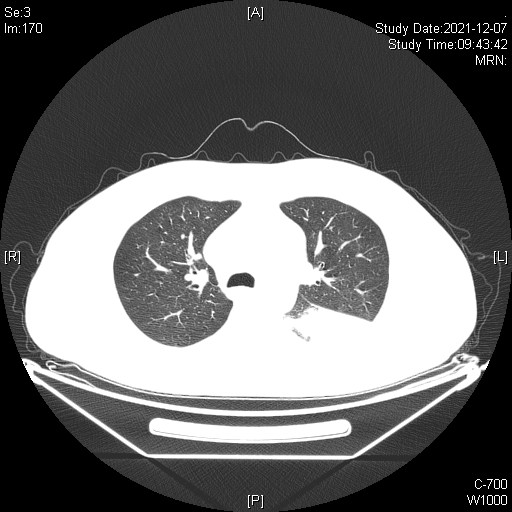

Supplement: Supplementary file 2 — Supplementary Information 2. [file 41598_2023_50702_MOESM2_ESM.zip › image data/OCAP/fan/fan (1).jpg]

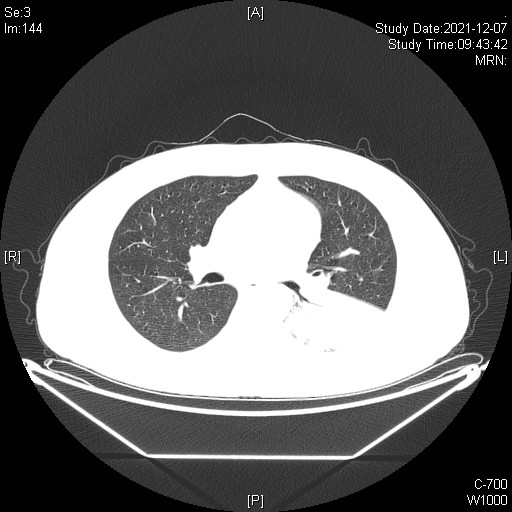

Supplement: Supplementary file 2 — Supplementary Information 2. [file 41598_2023_50702_MOESM2_ESM.zip › image data/OCAP/fan/fan (2).jpg]

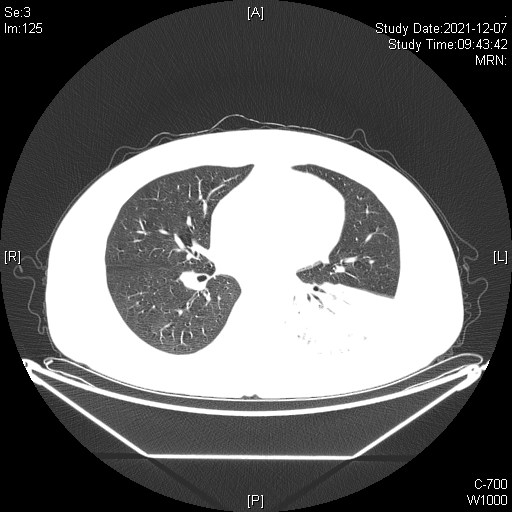

Supplement: Supplementary file 2 — Supplementary Information 2. [file 41598_2023_50702_MOESM2_ESM.zip › image data/OCAP/fan/fan (3).jpg]

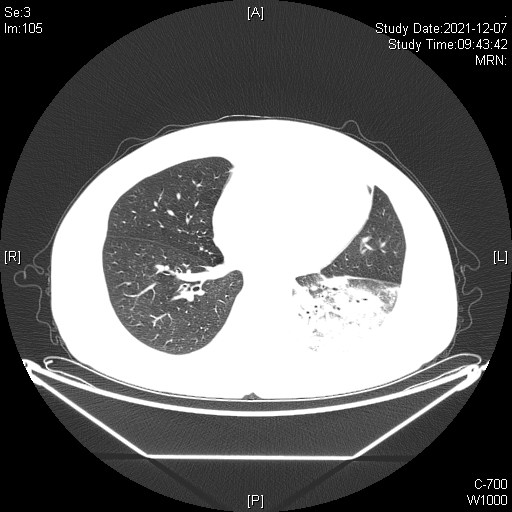

Supplement: Supplementary file 2 — Supplementary Information 2. [file 41598_2023_50702_MOESM2_ESM.zip › image data/OCAP/fan/fan (4).jpg]

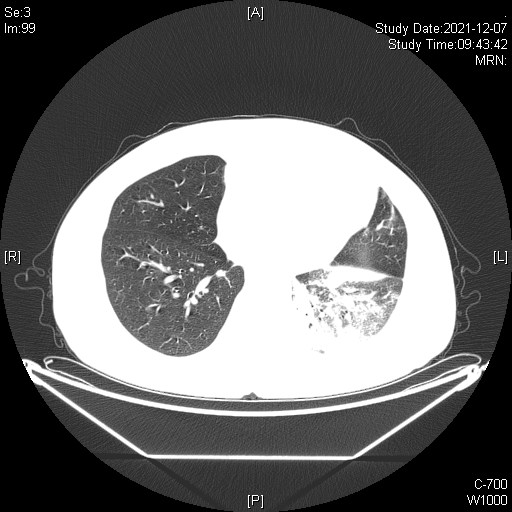

Supplement: Supplementary file 2 — Supplementary Information 2. [file 41598_2023_50702_MOESM2_ESM.zip › image data/OCAP/fan/fan (5).jpg]

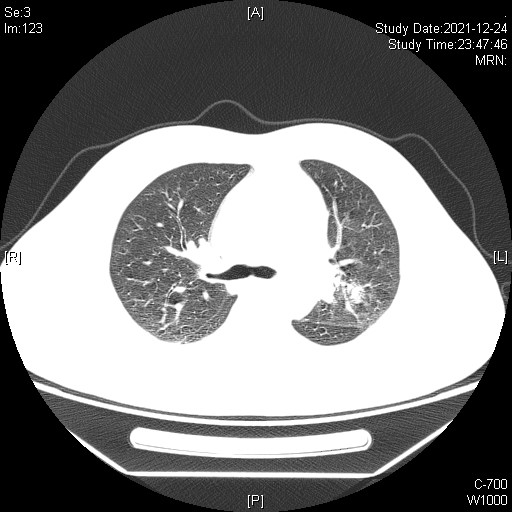

Supplement: Supplementary file 2 — Supplementary Information 2. [file 41598_2023_50702_MOESM2_ESM.zip › image data/OCAP/jia/jia (1).jpg]

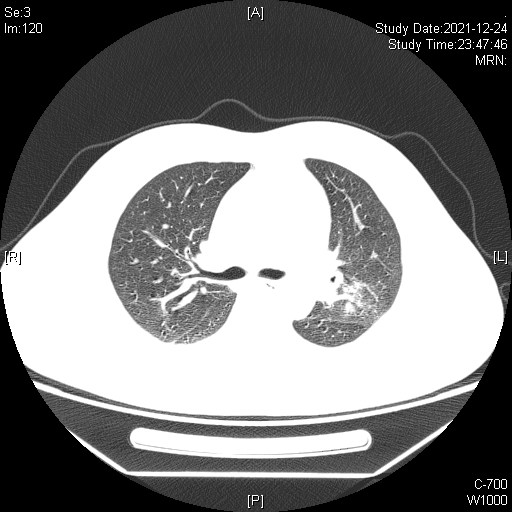

Supplement: Supplementary file 2 — Supplementary Information 2. [file 41598_2023_50702_MOESM2_ESM.zip › image data/OCAP/jia/jia (2).jpg]

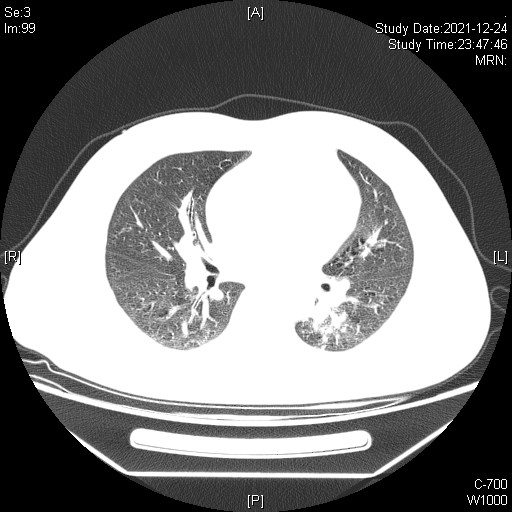

Supplement: Supplementary file 2 — Supplementary Information 2. [file 41598_2023_50702_MOESM2_ESM.zip › image data/OCAP/jia/jia (3).jpg]

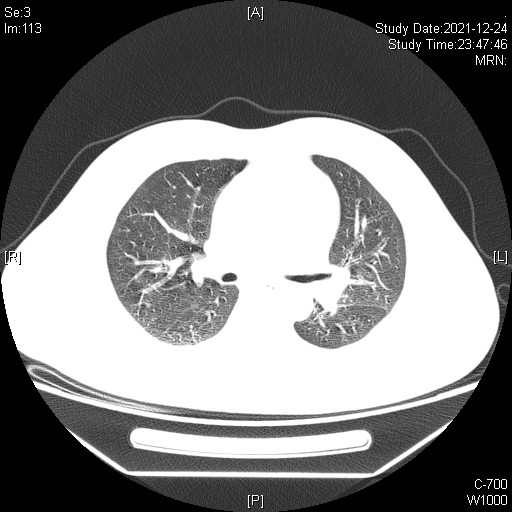

Supplement: Supplementary file 2 — Supplementary Information 2. [file 41598_2023_50702_MOESM2_ESM.zip › image data/OCAP/jia/jia (4).jpg]

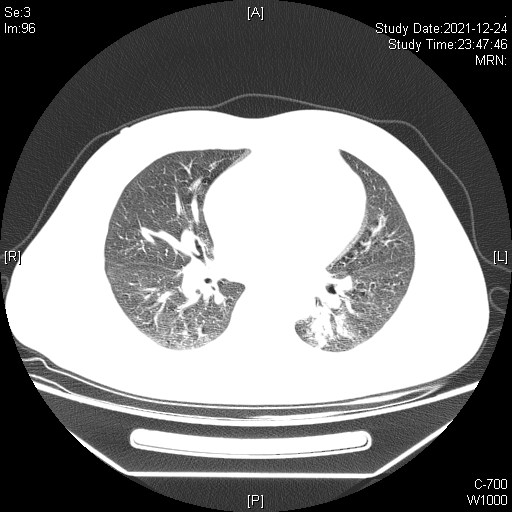

Supplement: Supplementary file 2 — Supplementary Information 2. [file 41598_2023_50702_MOESM2_ESM.zip › image data/OCAP/jia/jia (5).jpg]

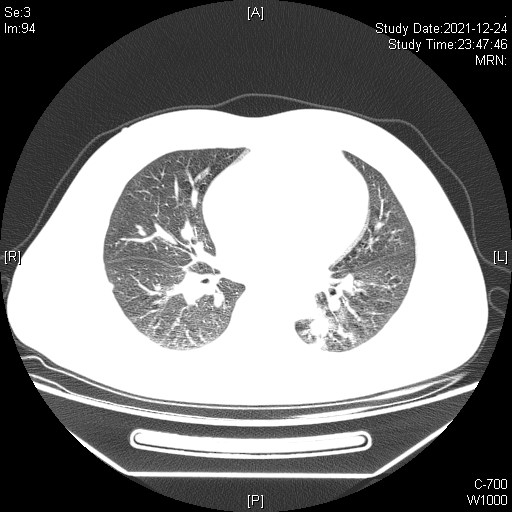

Supplement: Supplementary file 2 — Supplementary Information 2. [file 41598_2023_50702_MOESM2_ESM.zip › image data/OCAP/jia2/jia (1).jpg]

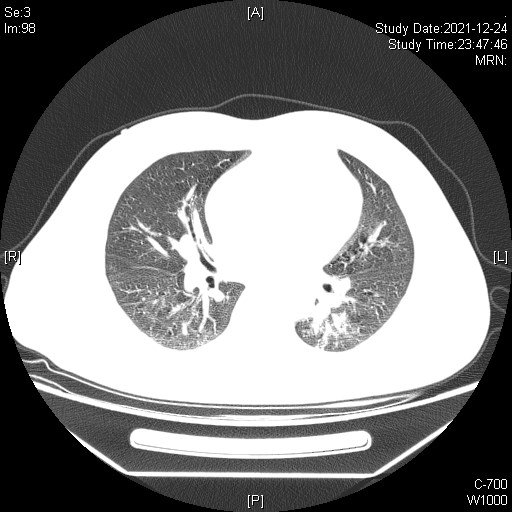

Supplement: Supplementary file 2 — Supplementary Information 2. [file 41598_2023_50702_MOESM2_ESM.zip › image data/OCAP/jia2/jia (2).jpg]

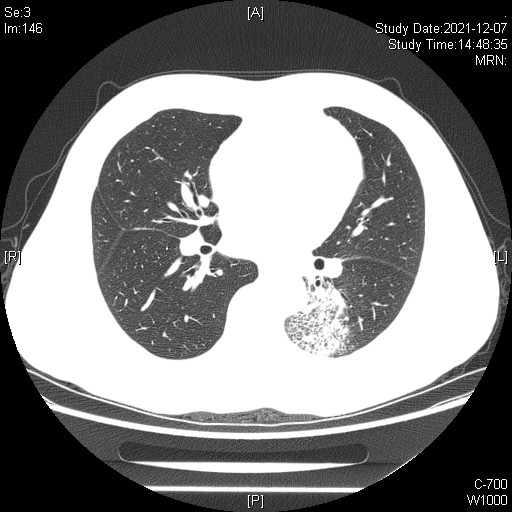

Supplement: Supplementary file 2 — Supplementary Information 2. [file 41598_2023_50702_MOESM2_ESM.zip › image data/OCAP/liu/liu (1).jpg]

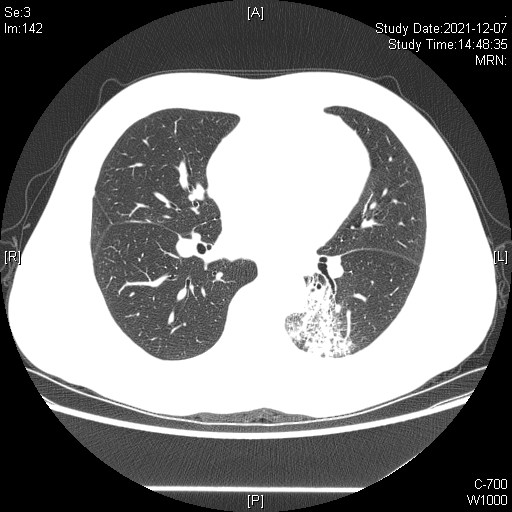

Supplement: Supplementary file 2 — Supplementary Information 2. [file 41598_2023_50702_MOESM2_ESM.zip › image data/OCAP/liu/liu (2).jpg]

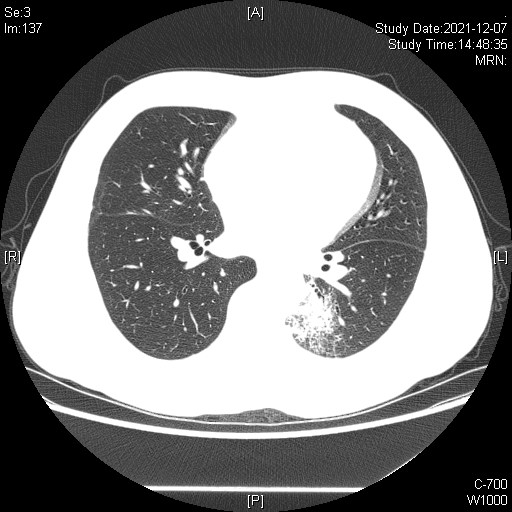

Supplement: Supplementary file 2 — Supplementary Information 2. [file 41598_2023_50702_MOESM2_ESM.zip › image data/OCAP/liu/liu (3).jpg]

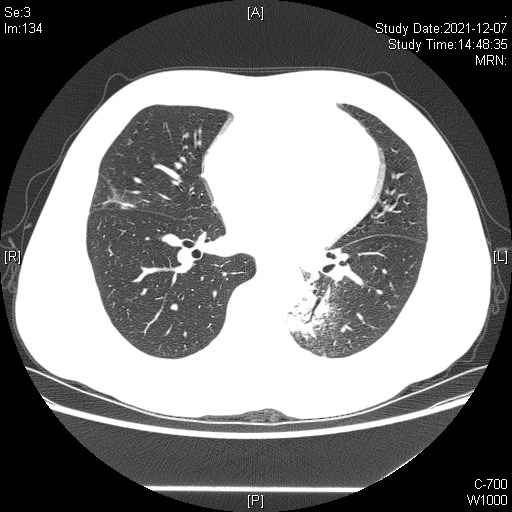

Supplement: Supplementary file 2 — Supplementary Information 2. [file 41598_2023_50702_MOESM2_ESM.zip › image data/OCAP/liu/liu (4).jpg]

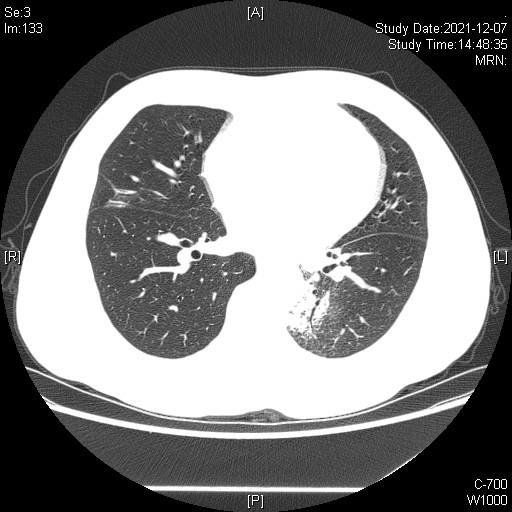

Supplement: Supplementary file 2 — Supplementary Information 2. [file 41598_2023_50702_MOESM2_ESM.zip › image data/OCAP/liu/liu (5).jpg]

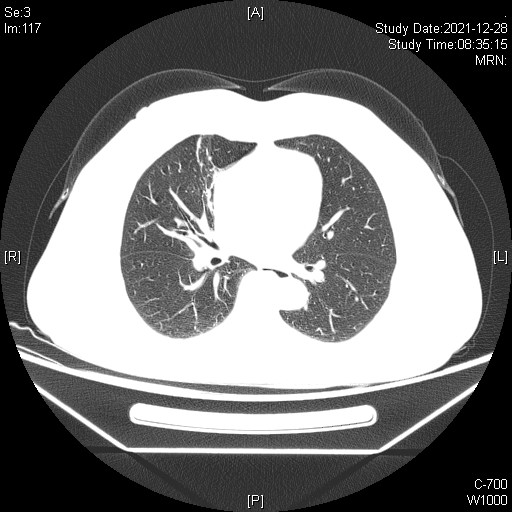

Supplement: Supplementary file 2 — Supplementary Information 2. [file 41598_2023_50702_MOESM2_ESM.zip › image data/OCAP/wang/wang (1).jpg]

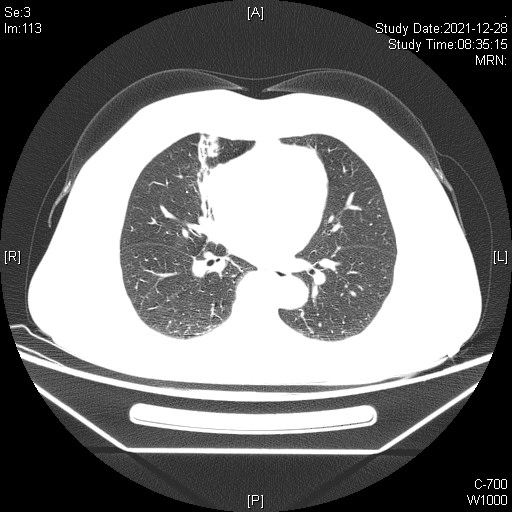

Supplement: Supplementary file 2 — Supplementary Information 2. [file 41598_2023_50702_MOESM2_ESM.zip › image data/OCAP/wang/wang (2).jpg]

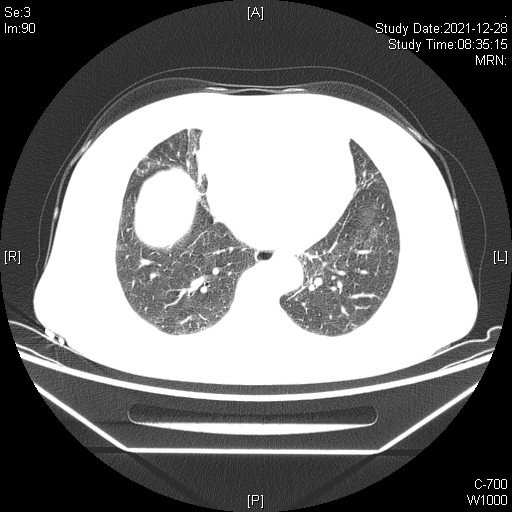

Supplement: Supplementary file 2 — Supplementary Information 2. [file 41598_2023_50702_MOESM2_ESM.zip › image data/OCAP/wang/wang (3).jpg]

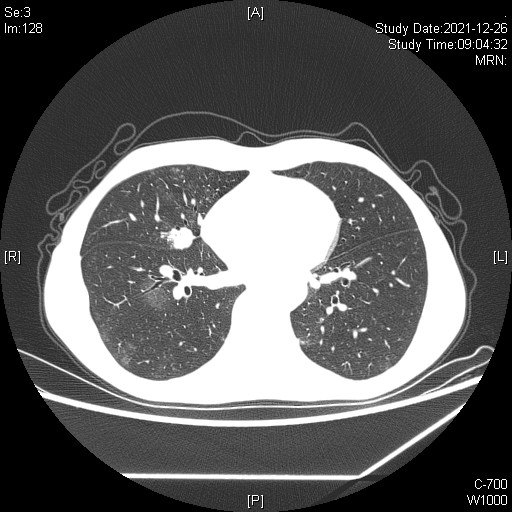

Supplement: Supplementary file 2 — Supplementary Information 2. [file 41598_2023_50702_MOESM2_ESM.zip › image data/OCAP/wang2/wang (1).jpg]

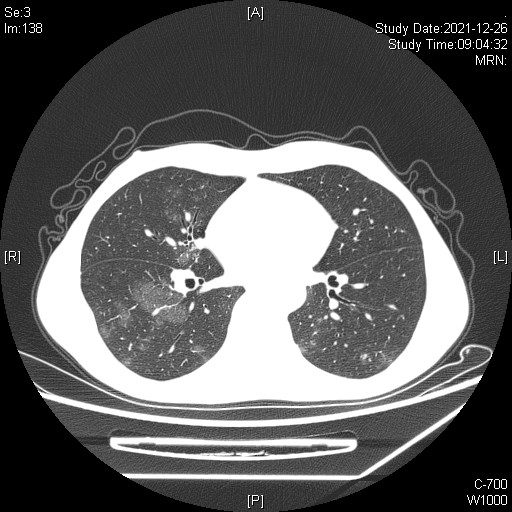

Supplement: Supplementary file 2 — Supplementary Information 2. [file 41598_2023_50702_MOESM2_ESM.zip › image data/OCAP/wang2/wang (2).jpg]

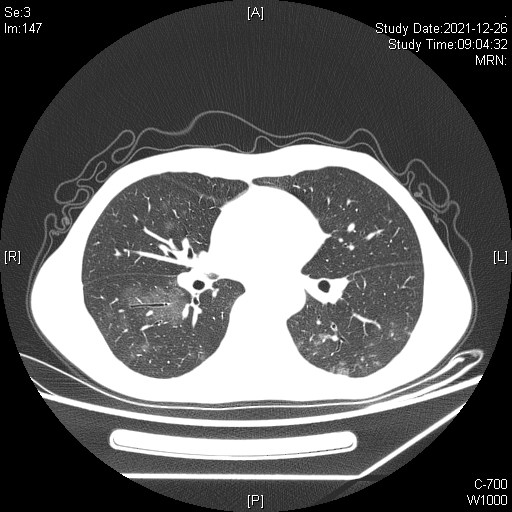

Supplement: Supplementary file 2 — Supplementary Information 2. [file 41598_2023_50702_MOESM2_ESM.zip › image data/OCAP/wang2/wang (3).jpg]

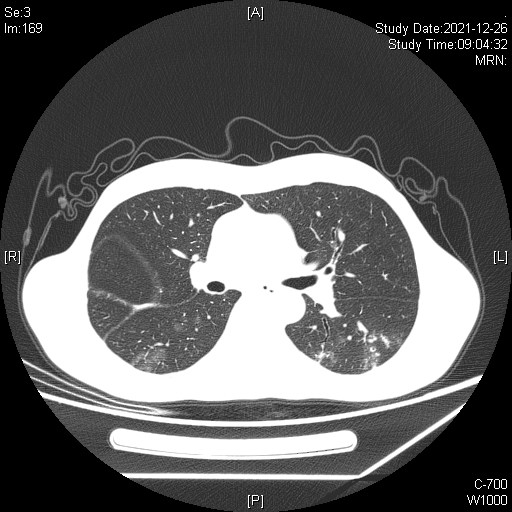

Supplement: Supplementary file 2 — Supplementary Information 2. [file 41598_2023_50702_MOESM2_ESM.zip › image data/OCAP/wang2/wang (4).jpg]

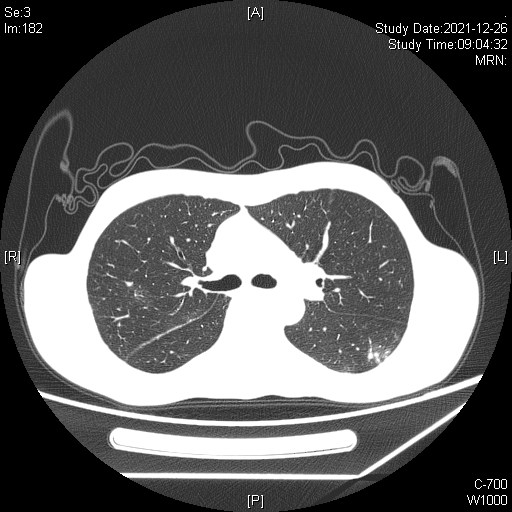

Supplement: Supplementary file 2 — Supplementary Information 2. [file 41598_2023_50702_MOESM2_ESM.zip › image data/OCAP/wang2/wang (5).jpg]

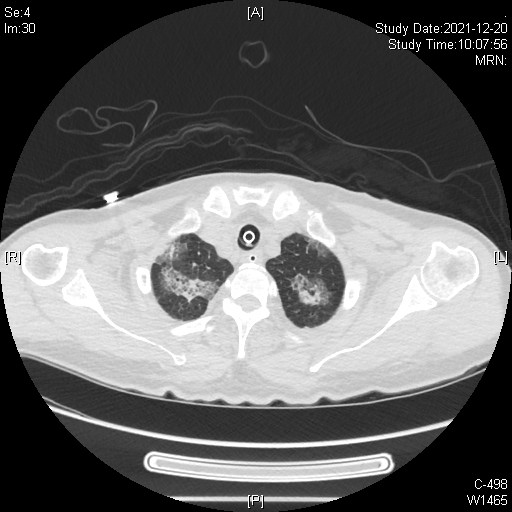

Supplement: Supplementary file 2 — Supplementary Information 2. [file 41598_2023_50702_MOESM2_ESM.zip › image data/OCAP/xu/xu (1).jpg]

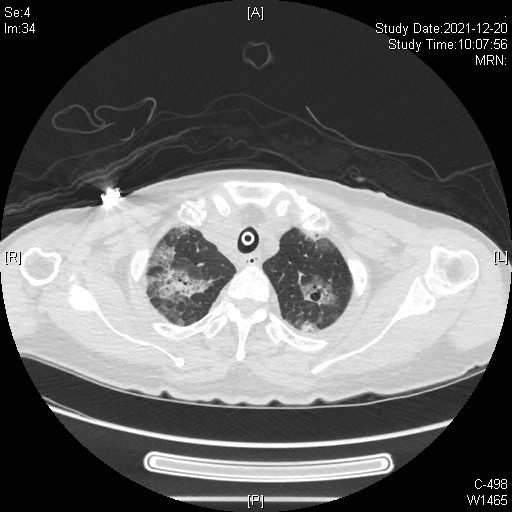

Supplement: Supplementary file 2 — Supplementary Information 2. [file 41598_2023_50702_MOESM2_ESM.zip › image data/OCAP/xu/xu (2).jpg]

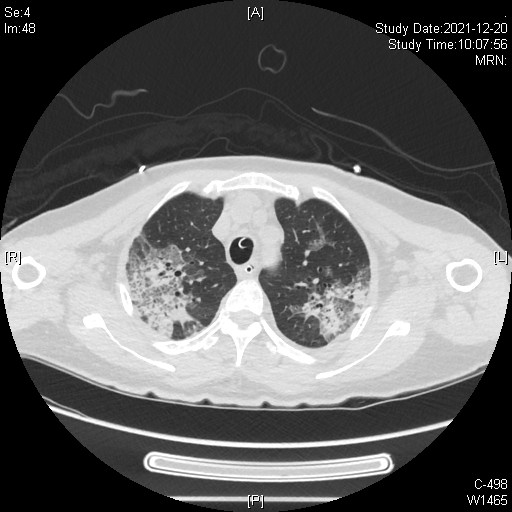

Supplement: Supplementary file 2 — Supplementary Information 2. [file 41598_2023_50702_MOESM2_ESM.zip › image data/OCAP/xu/xu (3).jpg]

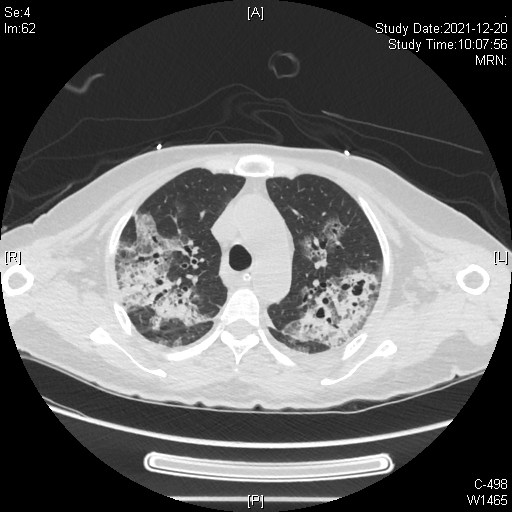

Supplement: Supplementary file 2 — Supplementary Information 2. [file 41598_2023_50702_MOESM2_ESM.zip › image data/OCAP/xu/xu (4).jpg]

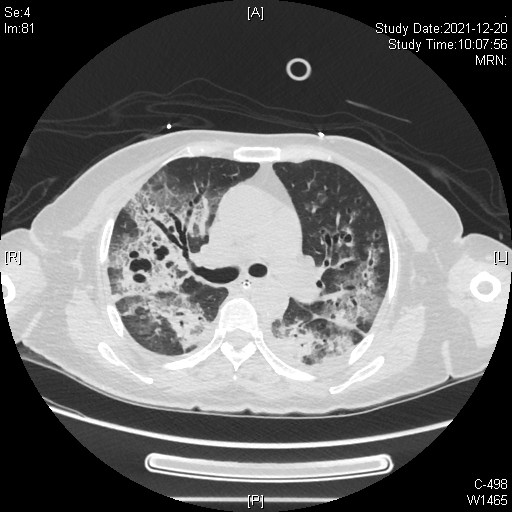

Supplement: Supplementary file 2 — Supplementary Information 2. [file 41598_2023_50702_MOESM2_ESM.zip › image data/OCAP/xu/xu (5).jpg]

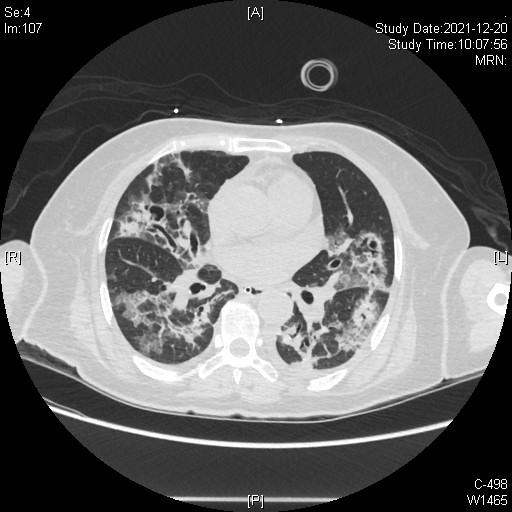

Supplement: Supplementary file 2 — Supplementary Information 2. [file 41598_2023_50702_MOESM2_ESM.zip › image data/OCAP/xu/xu (6).jpg]

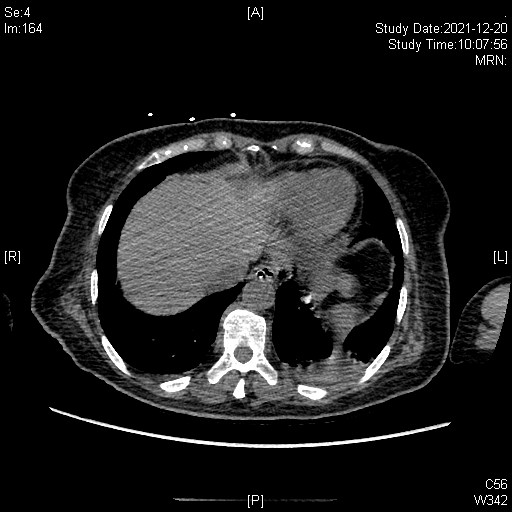

Supplement: Supplementary file 2 — Supplementary Information 2. [file 41598_2023_50702_MOESM2_ESM.zip › image data/OCAP/xu/xu (7).jpg]
