# Supplementary figures and images for: Diagnostic significance of HRCT imaging features in adult mycoplasma pneumonia: a retrospective study (part 1 of 2)
Source: Sci Rep. 2024 Jan 2;14:153. doi: 10.1038/s41598-023-50702-3 (PMC10761950; doi:10.1038/s41598-023-50702-3)

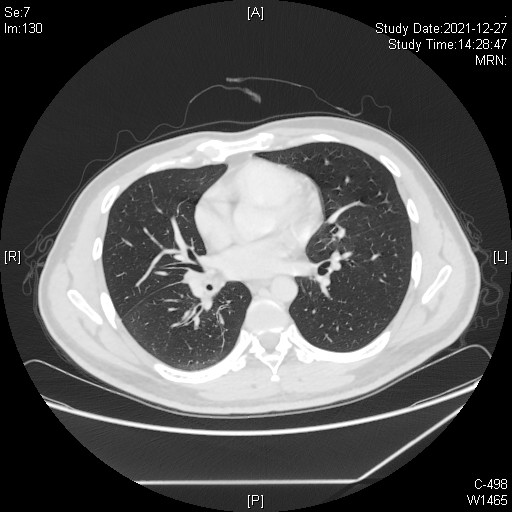

Supplement: Supplementary file 2 — Supplementary Information 2. [file 41598_2023_50702_MOESM2_ESM.zip › image data/MPP/cui/1.jpg]

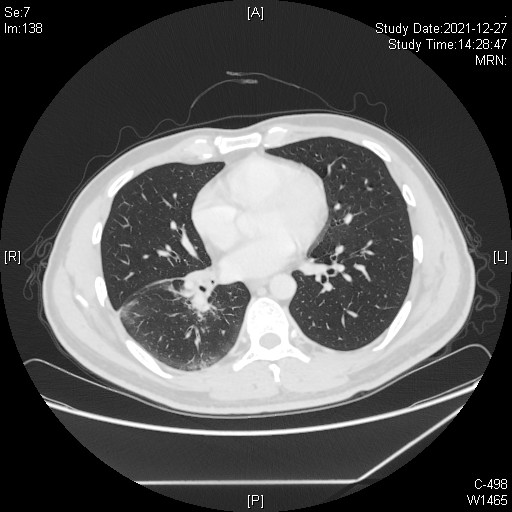

Supplement: Supplementary file 2 — Supplementary Information 2. [file 41598_2023_50702_MOESM2_ESM.zip › image data/MPP/cui/2.jpg]

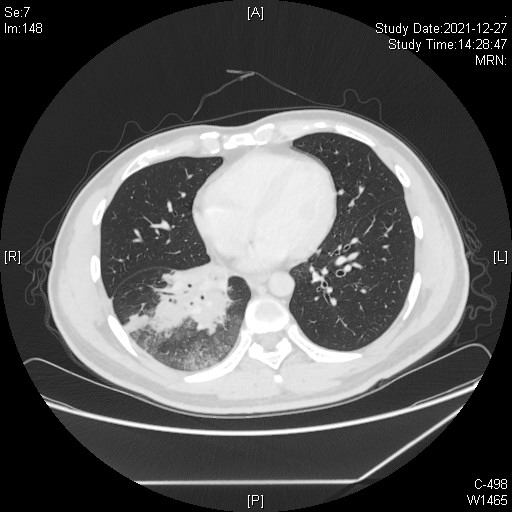

Supplement: Supplementary file 2 — Supplementary Information 2. [file 41598_2023_50702_MOESM2_ESM.zip › image data/MPP/cui/3.jpg]

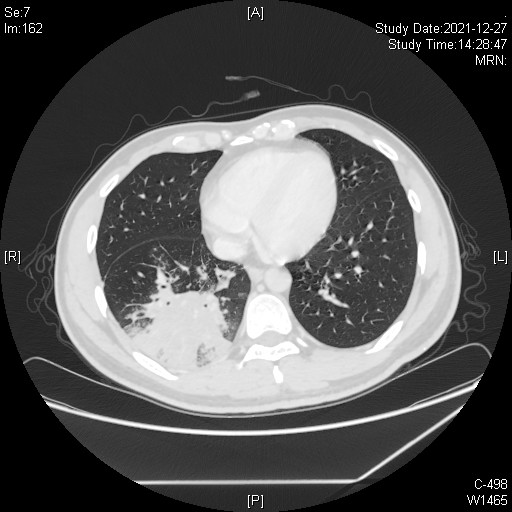

Supplement: Supplementary file 2 — Supplementary Information 2. [file 41598_2023_50702_MOESM2_ESM.zip › image data/MPP/cui/4.jpg]

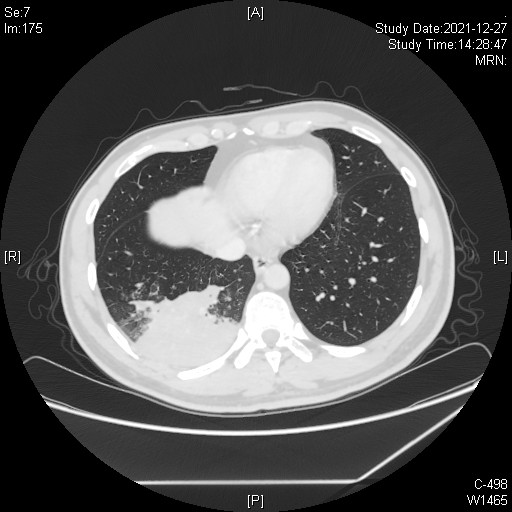

Supplement: Supplementary file 2 — Supplementary Information 2. [file 41598_2023_50702_MOESM2_ESM.zip › image data/MPP/cui/5.jpg]

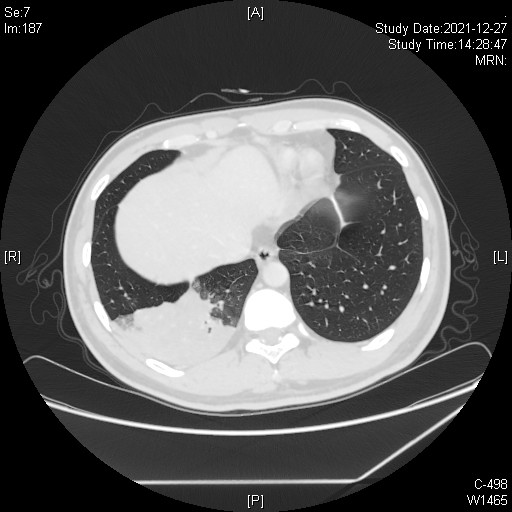

Supplement: Supplementary file 2 — Supplementary Information 2. [file 41598_2023_50702_MOESM2_ESM.zip › image data/MPP/cui/6.jpg]

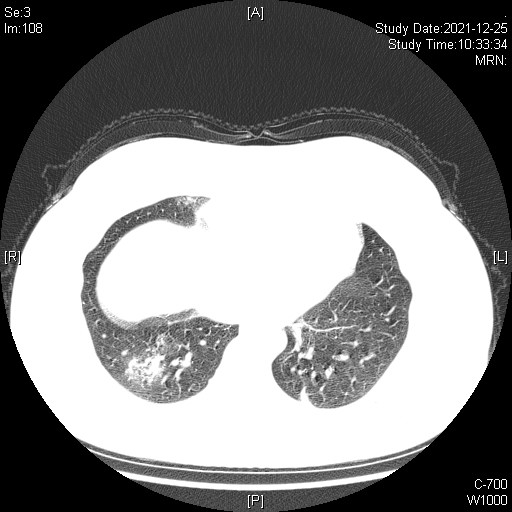

Supplement: Supplementary file 2 — Supplementary Information 2. [file 41598_2023_50702_MOESM2_ESM.zip › image data/MPP/du/1.jpg]

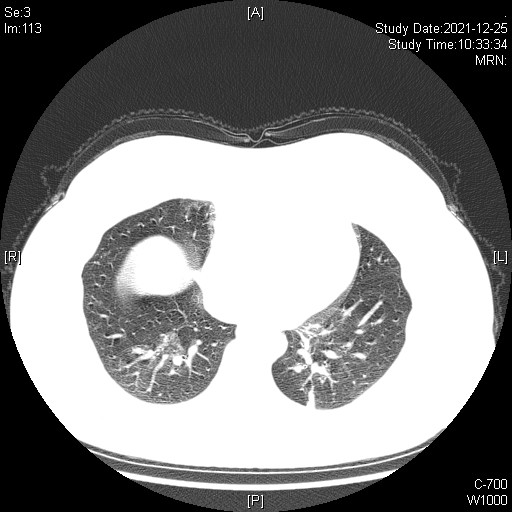

Supplement: Supplementary file 2 — Supplementary Information 2. [file 41598_2023_50702_MOESM2_ESM.zip › image data/MPP/du/2.jpg]

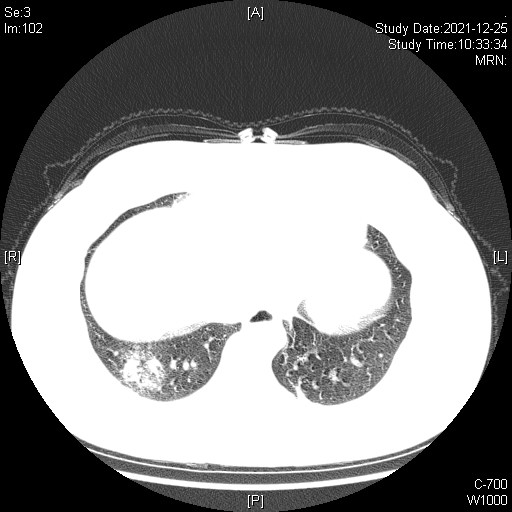

Supplement: Supplementary file 2 — Supplementary Information 2. [file 41598_2023_50702_MOESM2_ESM.zip › image data/MPP/du/3.jpg]

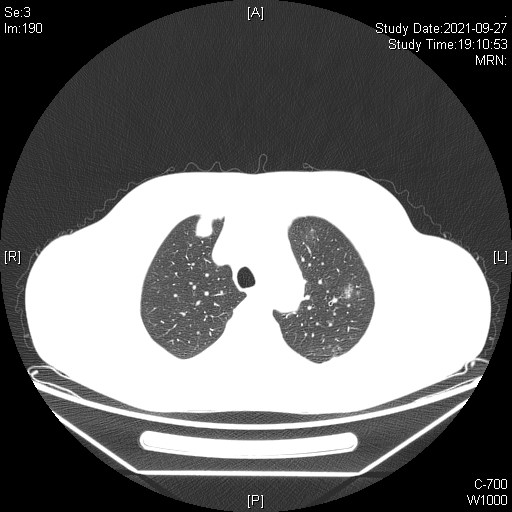

Supplement: Supplementary file 2 — Supplementary Information 2. [file 41598_2023_50702_MOESM2_ESM.zip › image data/MPP/fan/2021092701.jpg]

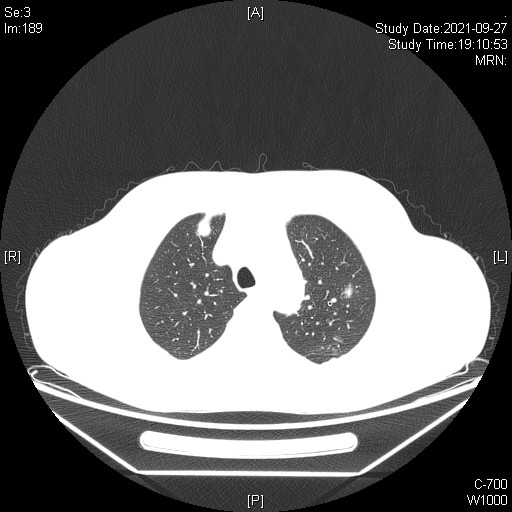

Supplement: Supplementary file 2 — Supplementary Information 2. [file 41598_2023_50702_MOESM2_ESM.zip › image data/MPP/fan/2021092702.jpg]

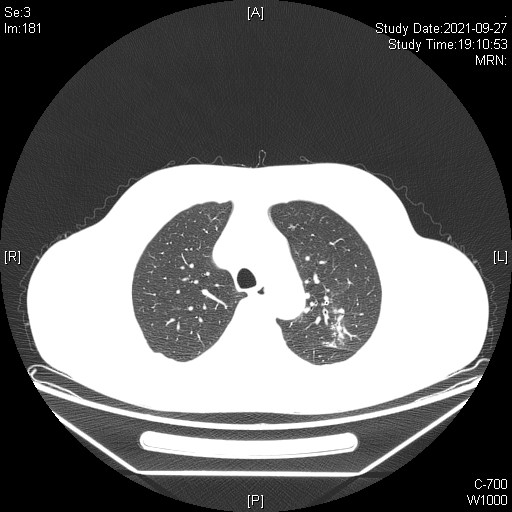

Supplement: Supplementary file 2 — Supplementary Information 2. [file 41598_2023_50702_MOESM2_ESM.zip › image data/MPP/fan/2021092703.jpg]

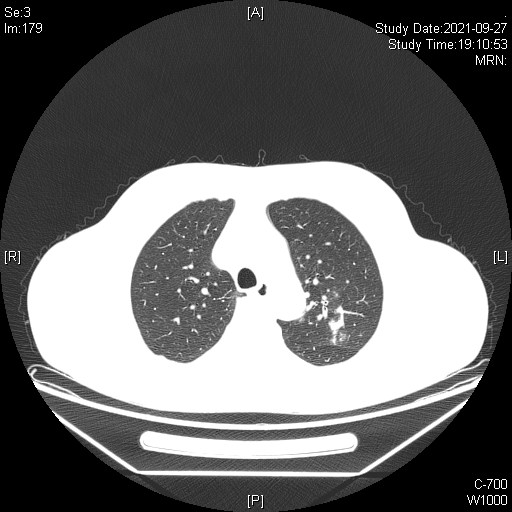

Supplement: Supplementary file 2 — Supplementary Information 2. [file 41598_2023_50702_MOESM2_ESM.zip › image data/MPP/fan/2021092704.jpg]

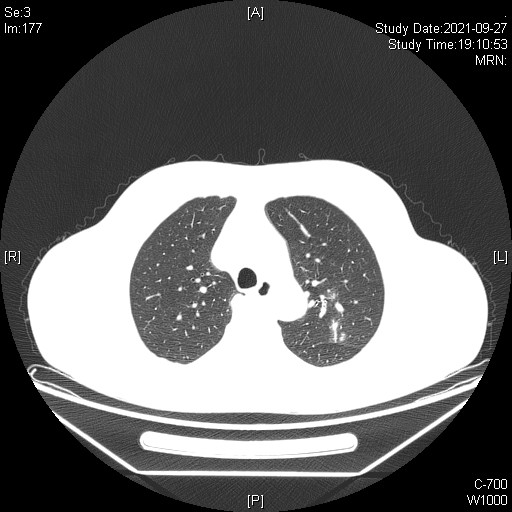

Supplement: Supplementary file 2 — Supplementary Information 2. [file 41598_2023_50702_MOESM2_ESM.zip › image data/MPP/fan/2021092705.jpg]

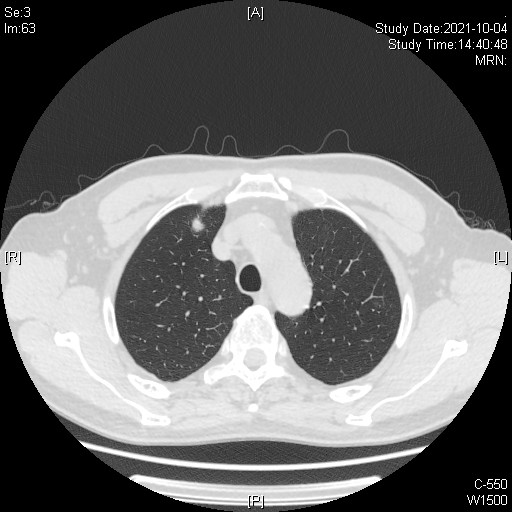

Supplement: Supplementary file 2 — Supplementary Information 2. [file 41598_2023_50702_MOESM2_ESM.zip › image data/MPP/fan/2021100401.jpg]

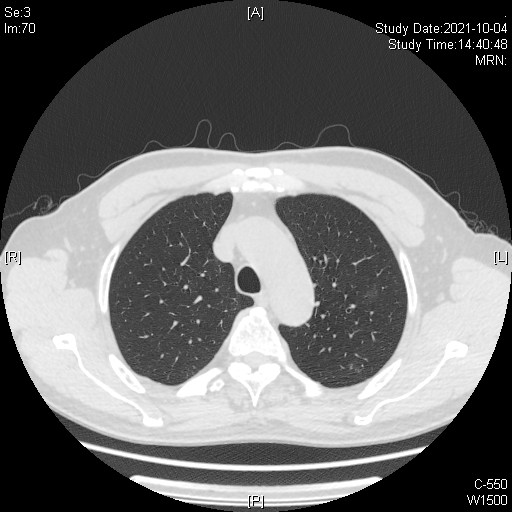

Supplement: Supplementary file 2 — Supplementary Information 2. [file 41598_2023_50702_MOESM2_ESM.zip › image data/MPP/fan/2021100402.jpg]

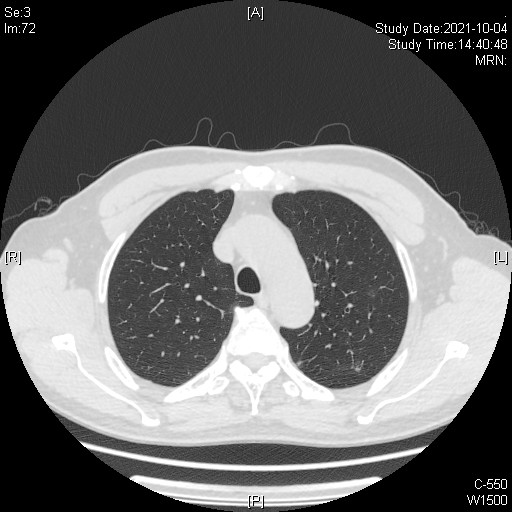

Supplement: Supplementary file 2 — Supplementary Information 2. [file 41598_2023_50702_MOESM2_ESM.zip › image data/MPP/fan/2021100403.jpg]

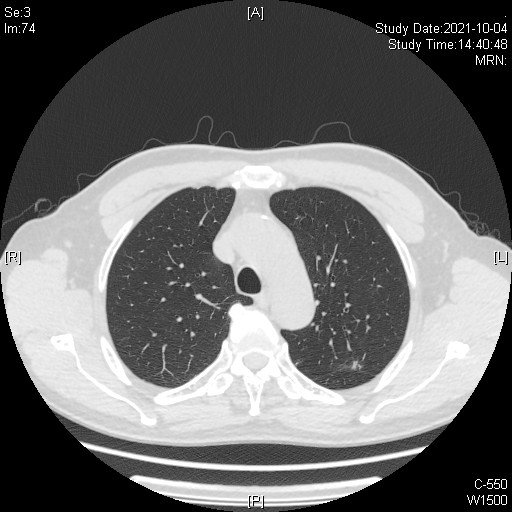

Supplement: Supplementary file 2 — Supplementary Information 2. [file 41598_2023_50702_MOESM2_ESM.zip › image data/MPP/fan/2021100404.jpg]

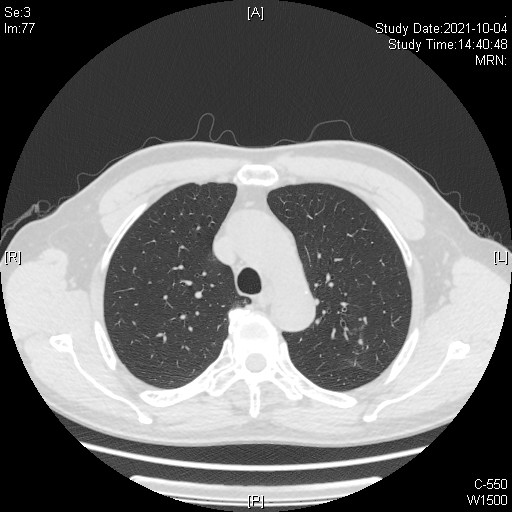

Supplement: Supplementary file 2 — Supplementary Information 2. [file 41598_2023_50702_MOESM2_ESM.zip › image data/MPP/fan/2021100405.jpg]

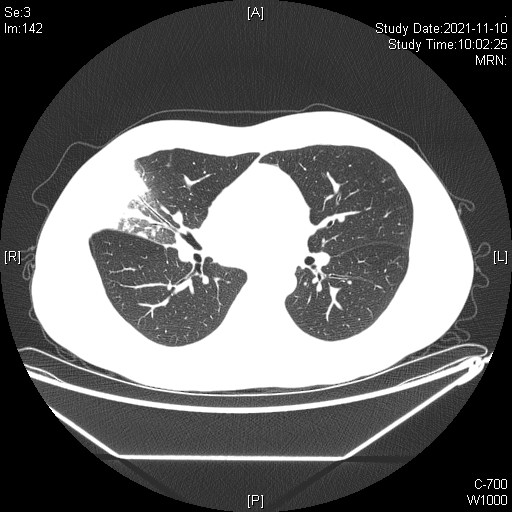

Supplement: Supplementary file 2 — Supplementary Information 2. [file 41598_2023_50702_MOESM2_ESM.zip › image data/MPP/he/1.jpg]

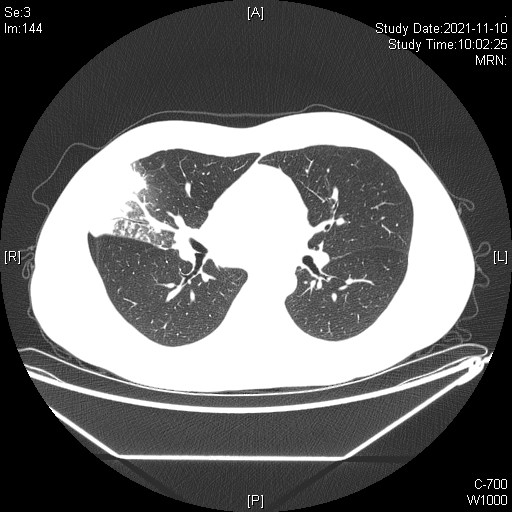

Supplement: Supplementary file 2 — Supplementary Information 2. [file 41598_2023_50702_MOESM2_ESM.zip › image data/MPP/he/2.jpg]

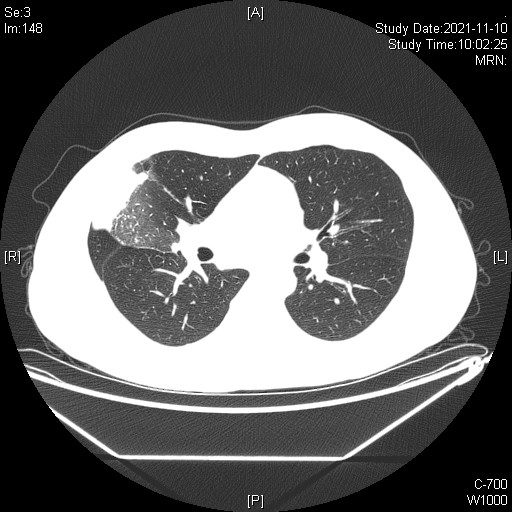

Supplement: Supplementary file 2 — Supplementary Information 2. [file 41598_2023_50702_MOESM2_ESM.zip › image data/MPP/he/3.jpg]

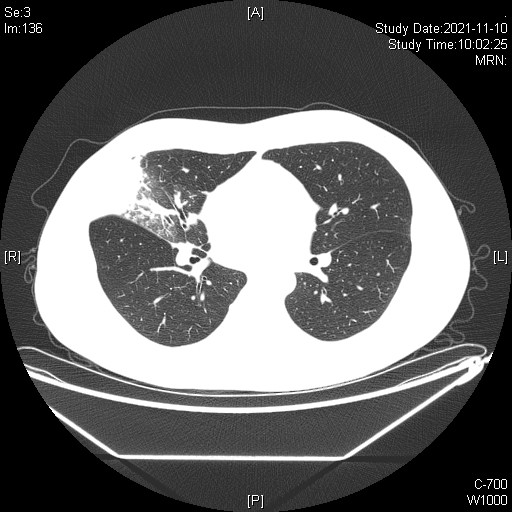

Supplement: Supplementary file 2 — Supplementary Information 2. [file 41598_2023_50702_MOESM2_ESM.zip › image data/MPP/he/4.jpg]

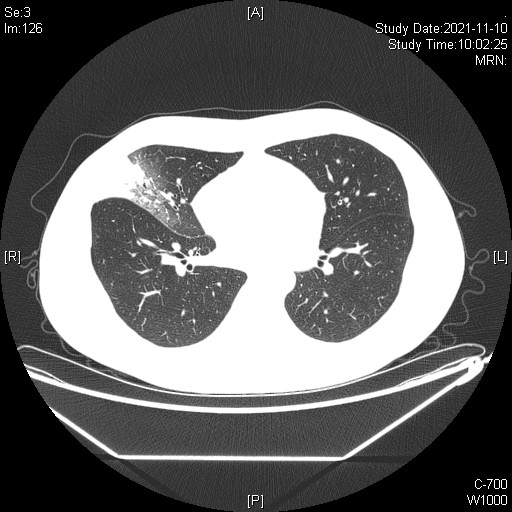

Supplement: Supplementary file 2 — Supplementary Information 2. [file 41598_2023_50702_MOESM2_ESM.zip › image data/MPP/he/5.jpg]

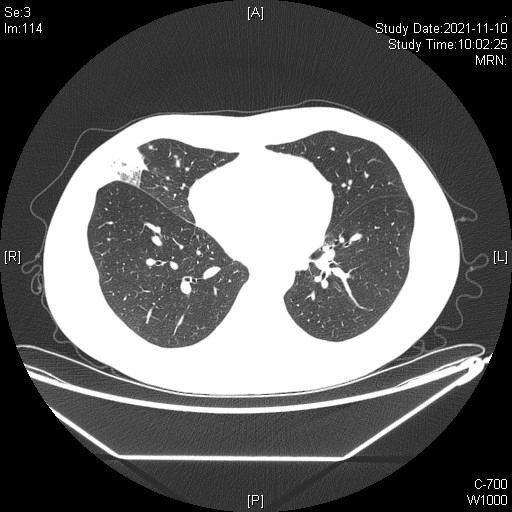

Supplement: Supplementary file 2 — Supplementary Information 2. [file 41598_2023_50702_MOESM2_ESM.zip › image data/MPP/he/6.jpg]

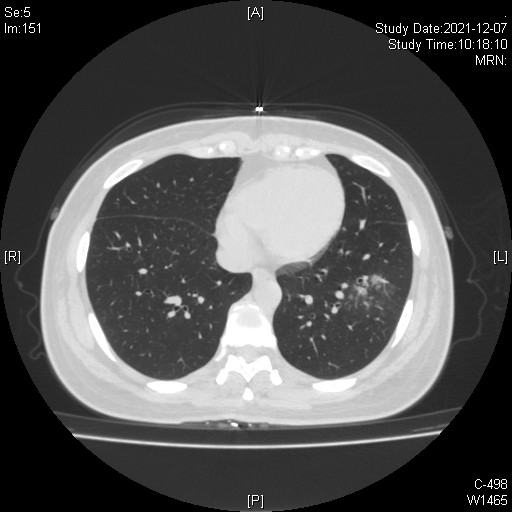

Supplement: Supplementary file 2 — Supplementary Information 2. [file 41598_2023_50702_MOESM2_ESM.zip › image data/MPP/li/1.jpg]

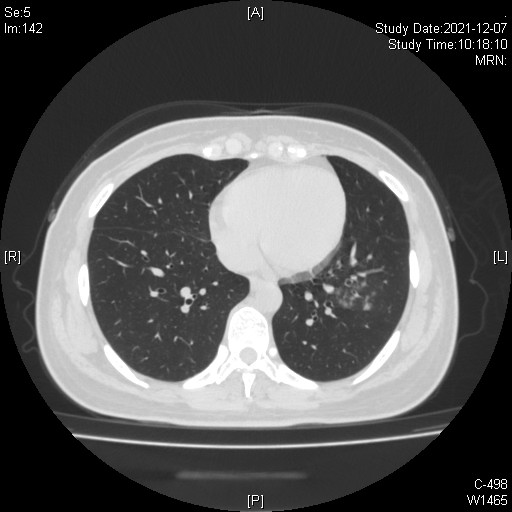

Supplement: Supplementary file 2 — Supplementary Information 2. [file 41598_2023_50702_MOESM2_ESM.zip › image data/MPP/li/2.jpg]

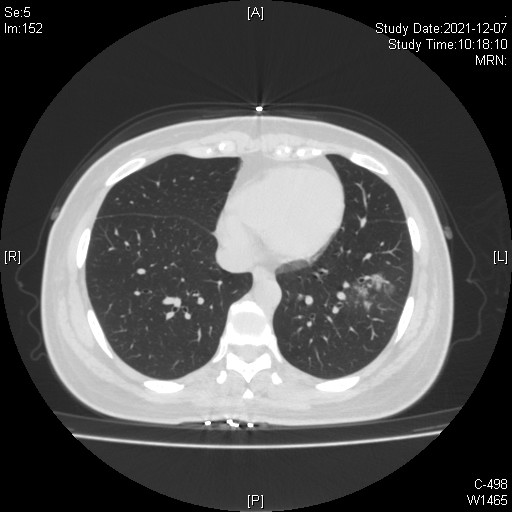

Supplement: Supplementary file 2 — Supplementary Information 2. [file 41598_2023_50702_MOESM2_ESM.zip › image data/MPP/li/3.jpg]

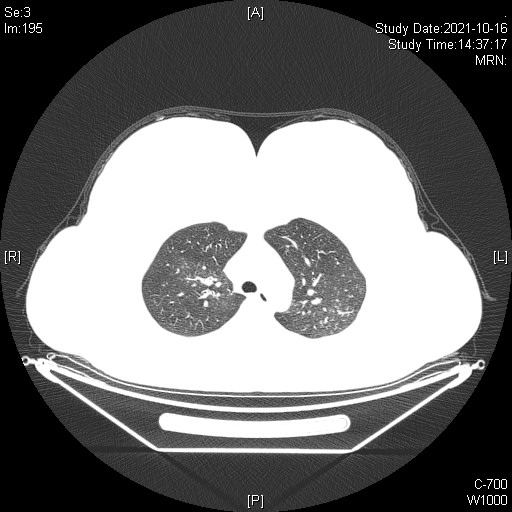

Supplement: Supplementary file 2 — Supplementary Information 2. [file 41598_2023_50702_MOESM2_ESM.zip › image data/MPP/li2/1.jpg]

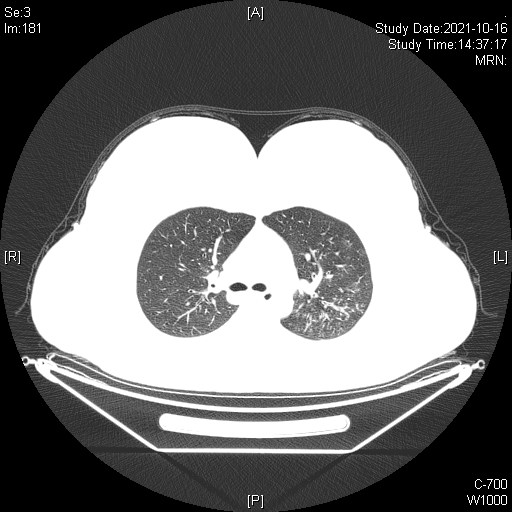

Supplement: Supplementary file 2 — Supplementary Information 2. [file 41598_2023_50702_MOESM2_ESM.zip › image data/MPP/li2/2.jpg]

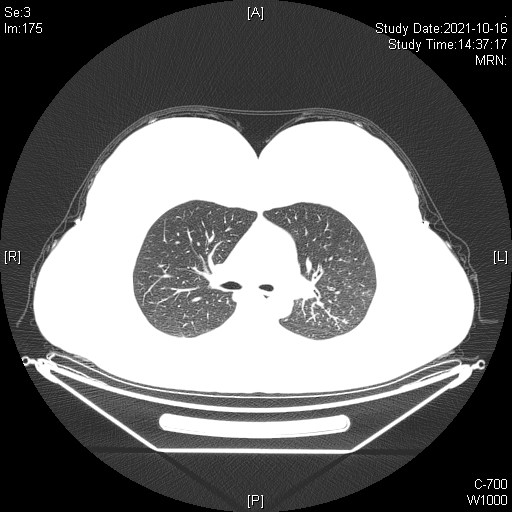

Supplement: Supplementary file 2 — Supplementary Information 2. [file 41598_2023_50702_MOESM2_ESM.zip › image data/MPP/li2/3.jpg]

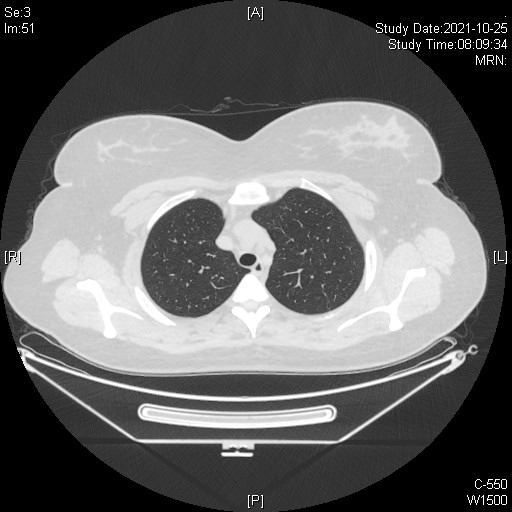

Supplement: Supplementary file 2 — Supplementary Information 2. [file 41598_2023_50702_MOESM2_ESM.zip › image data/MPP/li2/4.jpg]

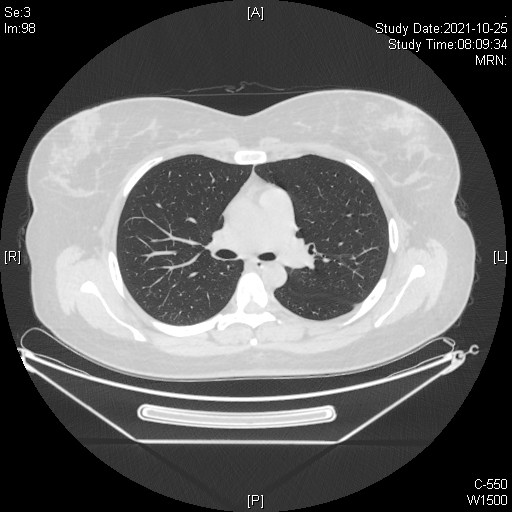

Supplement: Supplementary file 2 — Supplementary Information 2. [file 41598_2023_50702_MOESM2_ESM.zip › image data/MPP/li2/5.jpg]

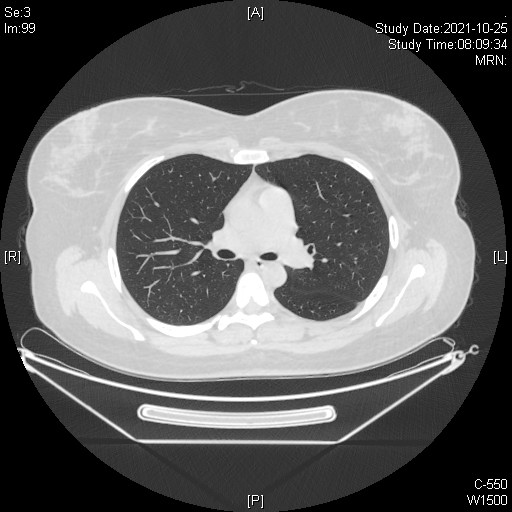

Supplement: Supplementary file 2 — Supplementary Information 2. [file 41598_2023_50702_MOESM2_ESM.zip › image data/MPP/li2/6.jpg]

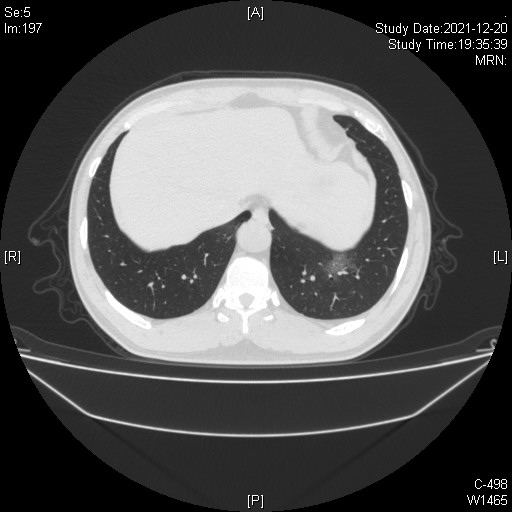

Supplement: Supplementary file 2 — Supplementary Information 2. [file 41598_2023_50702_MOESM2_ESM.zip › image data/MPP/meng/1.jpg]

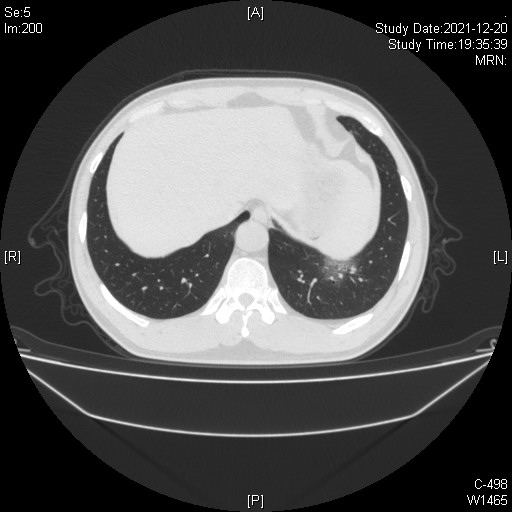

Supplement: Supplementary file 2 — Supplementary Information 2. [file 41598_2023_50702_MOESM2_ESM.zip › image data/MPP/meng/2.jpg]

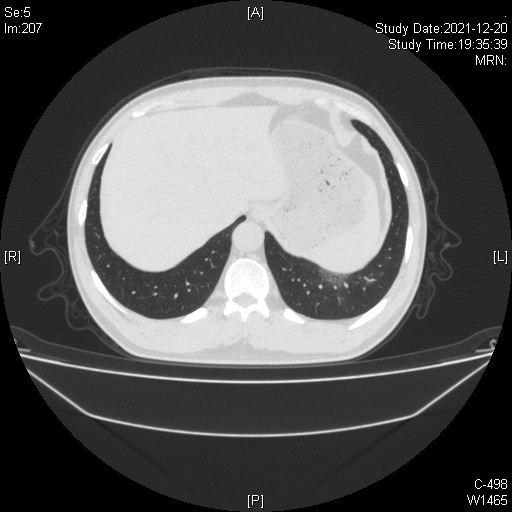

Supplement: Supplementary file 2 — Supplementary Information 2. [file 41598_2023_50702_MOESM2_ESM.zip › image data/MPP/meng/3.jpg]

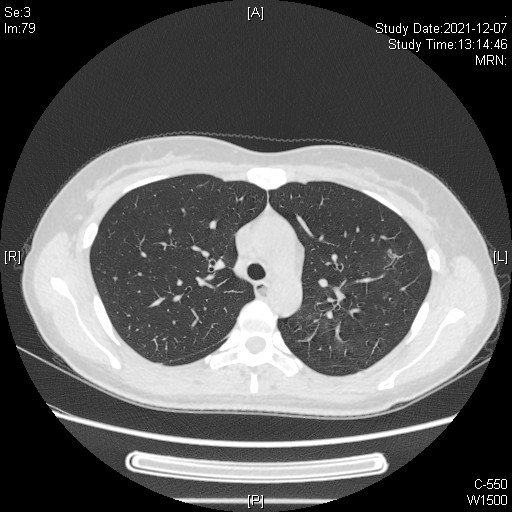

Supplement: Supplementary file 2 — Supplementary Information 2. [file 41598_2023_50702_MOESM2_ESM.zip › image data/MPP/qian/1.jpg]

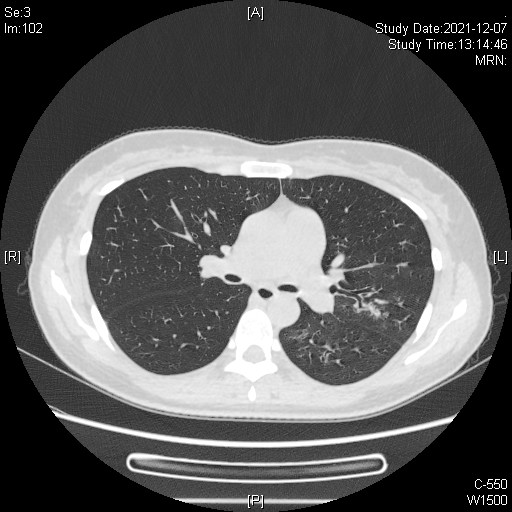

Supplement: Supplementary file 2 — Supplementary Information 2. [file 41598_2023_50702_MOESM2_ESM.zip › image data/MPP/qian/2.jpg]

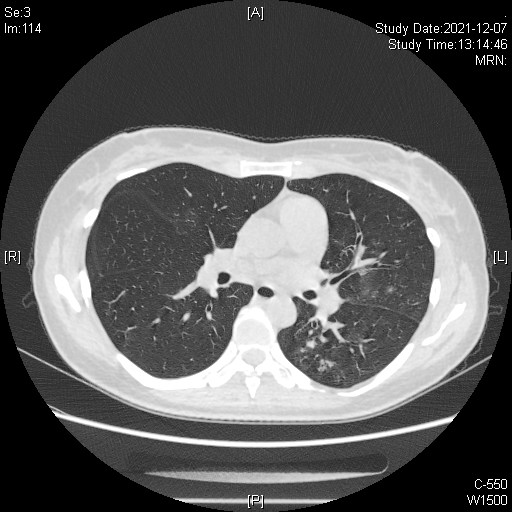

Supplement: Supplementary file 2 — Supplementary Information 2. [file 41598_2023_50702_MOESM2_ESM.zip › image data/MPP/qian/3.jpg]

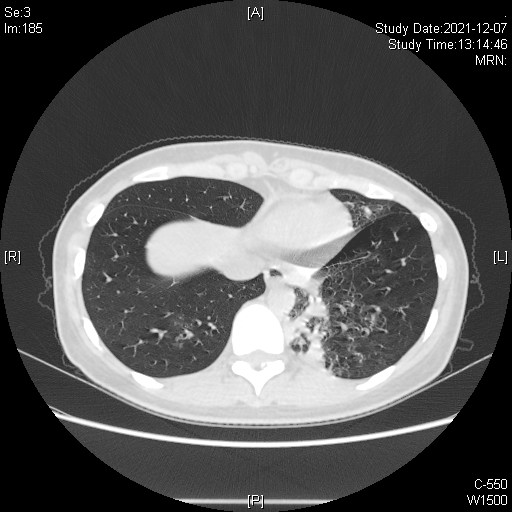

Supplement: Supplementary file 2 — Supplementary Information 2. [file 41598_2023_50702_MOESM2_ESM.zip › image data/MPP/qian/4.jpg]

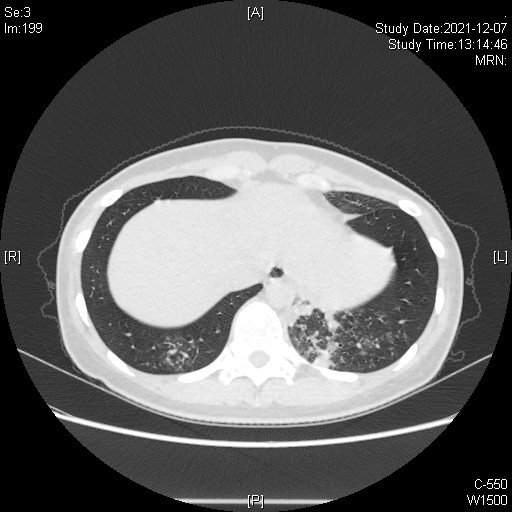

Supplement: Supplementary file 2 — Supplementary Information 2. [file 41598_2023_50702_MOESM2_ESM.zip › image data/MPP/qian/5.jpg]

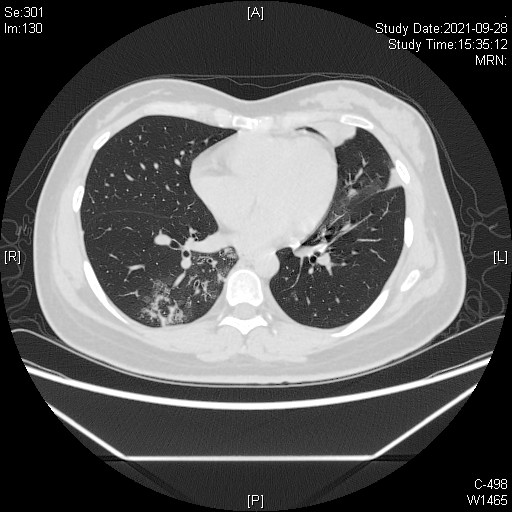

Supplement: Supplementary file 2 — Supplementary Information 2. [file 41598_2023_50702_MOESM2_ESM.zip › image data/MPP/song/2021092801.jpg]

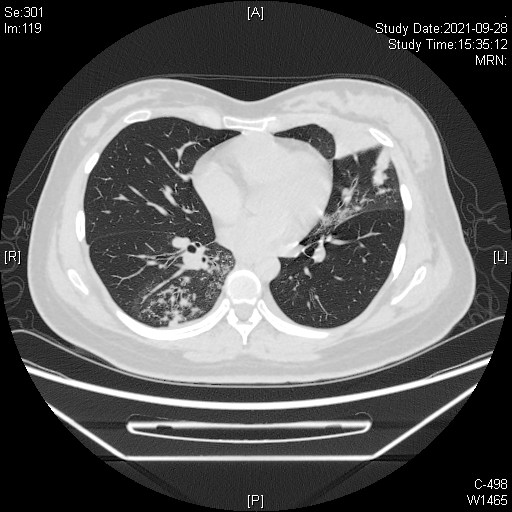

Supplement: Supplementary file 2 — Supplementary Information 2. [file 41598_2023_50702_MOESM2_ESM.zip › image data/MPP/song/2021092802.jpg]

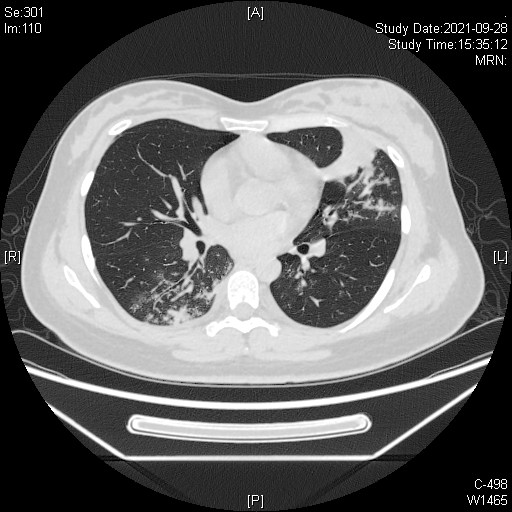

Supplement: Supplementary file 2 — Supplementary Information 2. [file 41598_2023_50702_MOESM2_ESM.zip › image data/MPP/song/2021092803.jpg]

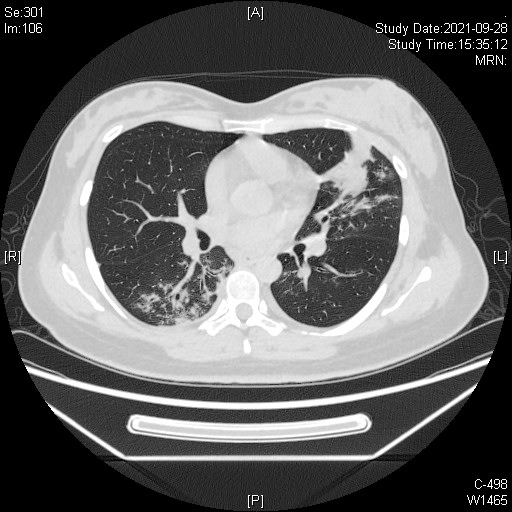

Supplement: Supplementary file 2 — Supplementary Information 2. [file 41598_2023_50702_MOESM2_ESM.zip › image data/MPP/song/2021092804.jpg]

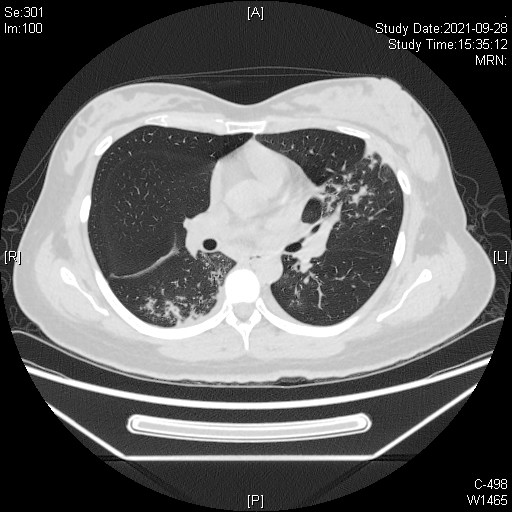

Supplement: Supplementary file 2 — Supplementary Information 2. [file 41598_2023_50702_MOESM2_ESM.zip › image data/MPP/song/2021092805.jpg]

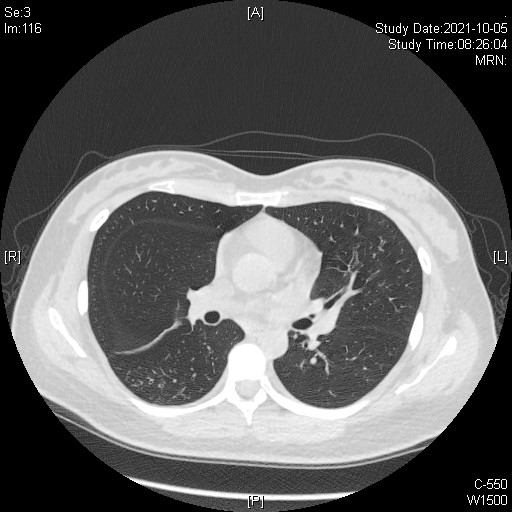

Supplement: Supplementary file 2 — Supplementary Information 2. [file 41598_2023_50702_MOESM2_ESM.zip › image data/MPP/song/2021100501.jpg]

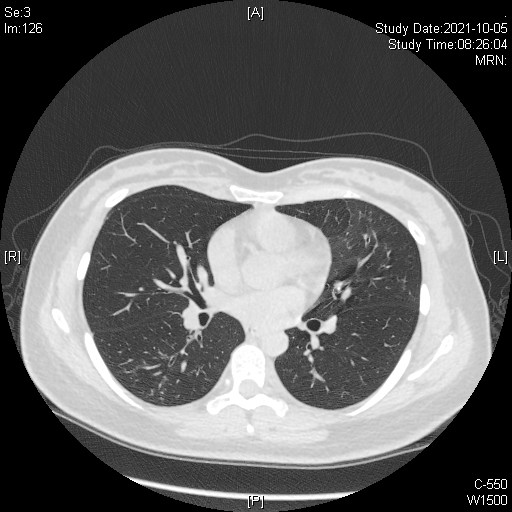

Supplement: Supplementary file 2 — Supplementary Information 2. [file 41598_2023_50702_MOESM2_ESM.zip › image data/MPP/song/2021100502.jpg]

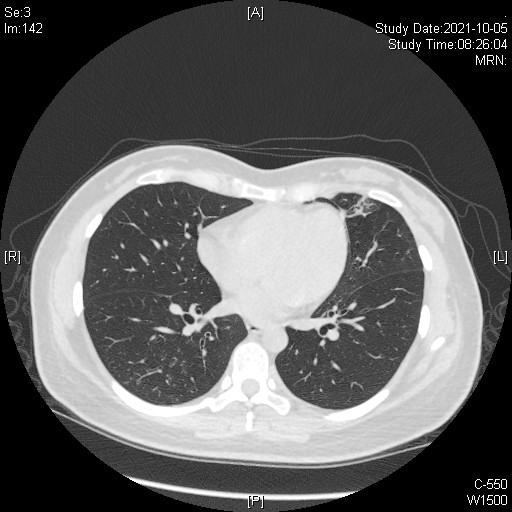

Supplement: Supplementary file 2 — Supplementary Information 2. [file 41598_2023_50702_MOESM2_ESM.zip › image data/MPP/song/2021100503.jpg]

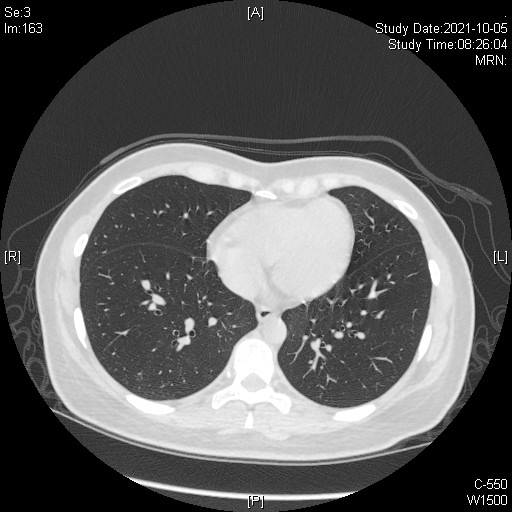

Supplement: Supplementary file 2 — Supplementary Information 2. [file 41598_2023_50702_MOESM2_ESM.zip › image data/MPP/song/2021100504.jpg]

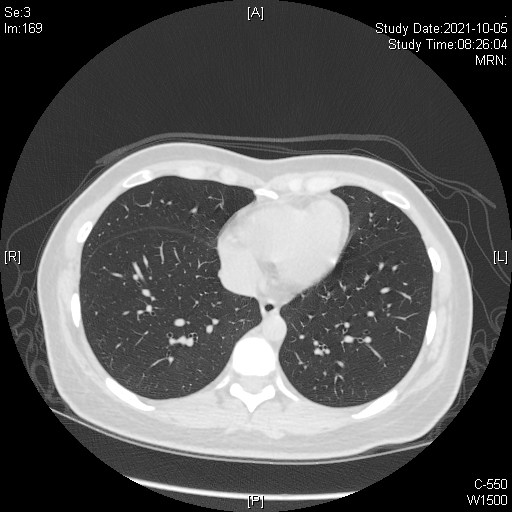

Supplement: Supplementary file 2 — Supplementary Information 2. [file 41598_2023_50702_MOESM2_ESM.zip › image data/MPP/song/2021100505.jpg]

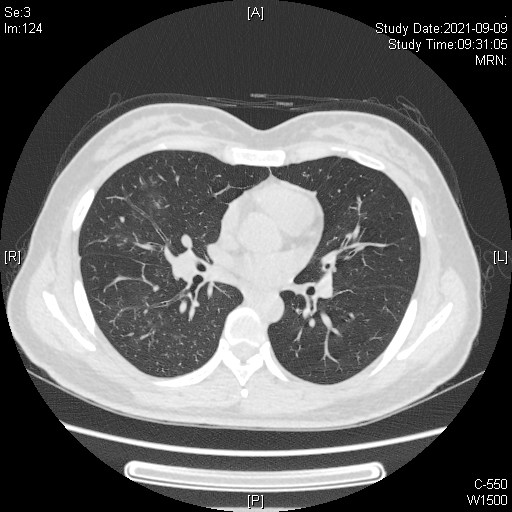

Supplement: Supplementary file 2 — Supplementary Information 2. [file 41598_2023_50702_MOESM2_ESM.zip › image data/MPP/wang/2021090901.jpg]

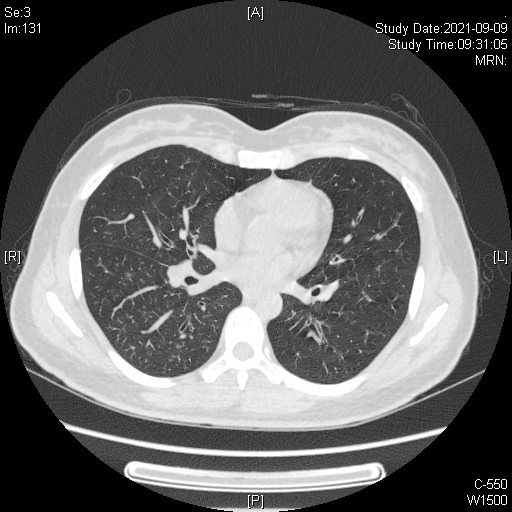

Supplement: Supplementary file 2 — Supplementary Information 2. [file 41598_2023_50702_MOESM2_ESM.zip › image data/MPP/wang/2021090902.jpg]

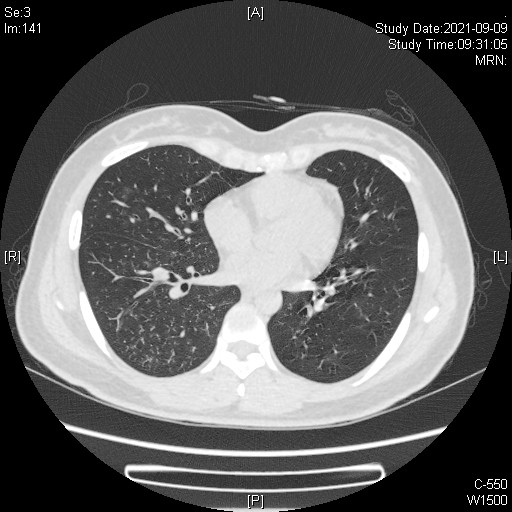

Supplement: Supplementary file 2 — Supplementary Information 2. [file 41598_2023_50702_MOESM2_ESM.zip › image data/MPP/wang/2021090903.jpg]

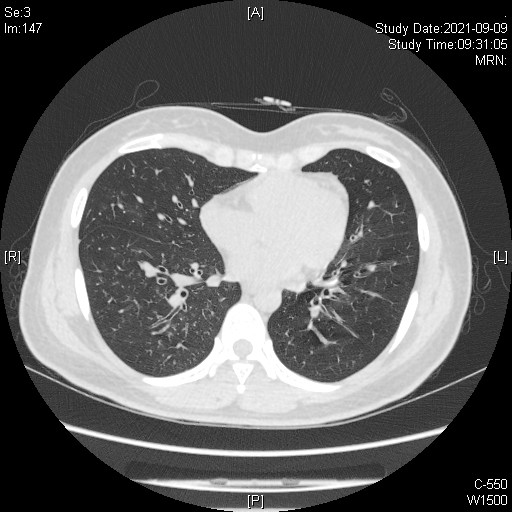

Supplement: Supplementary file 2 — Supplementary Information 2. [file 41598_2023_50702_MOESM2_ESM.zip › image data/MPP/wang/2021090904.jpg]

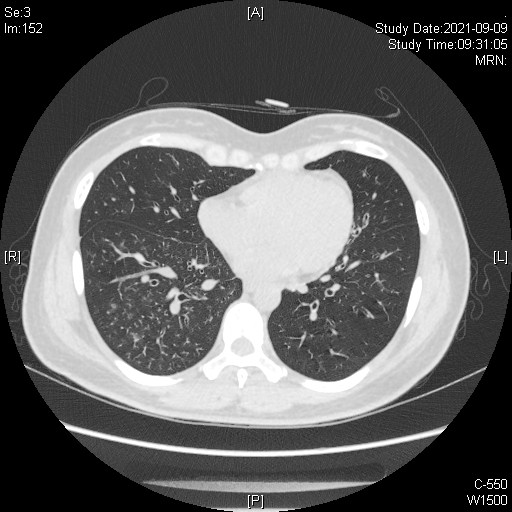

Supplement: Supplementary file 2 — Supplementary Information 2. [file 41598_2023_50702_MOESM2_ESM.zip › image data/MPP/wang/2021090905.jpg]

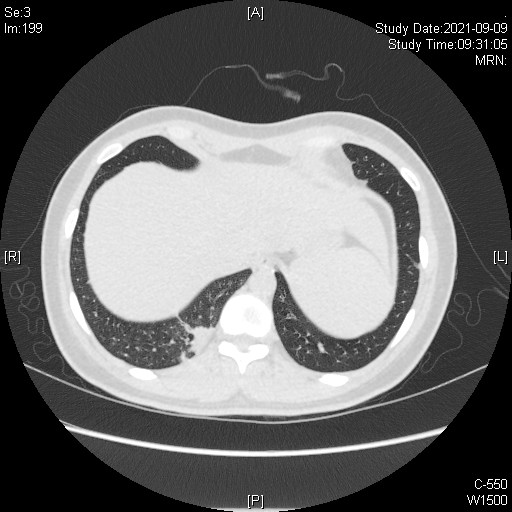

Supplement: Supplementary file 2 — Supplementary Information 2. [file 41598_2023_50702_MOESM2_ESM.zip › image data/MPP/wang/2021090906.jpg]

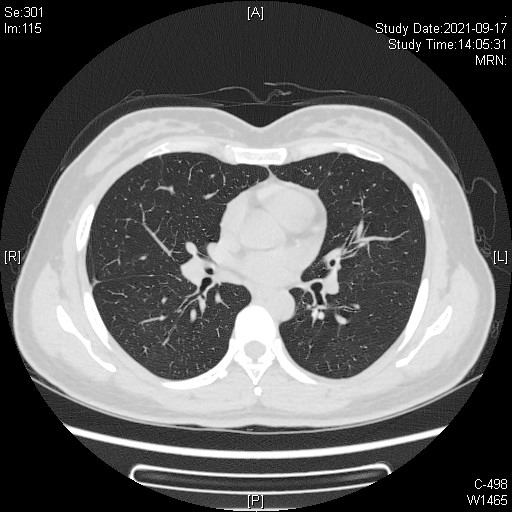

Supplement: Supplementary file 2 — Supplementary Information 2. [file 41598_2023_50702_MOESM2_ESM.zip › image data/MPP/wang/2021091701.jpg]

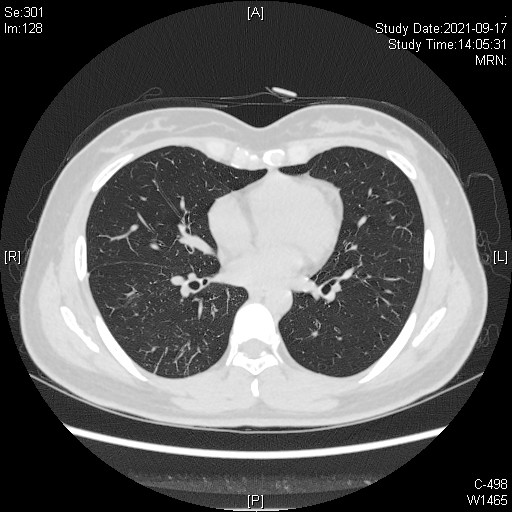

Supplement: Supplementary file 2 — Supplementary Information 2. [file 41598_2023_50702_MOESM2_ESM.zip › image data/MPP/wang/2021091702.jpg]

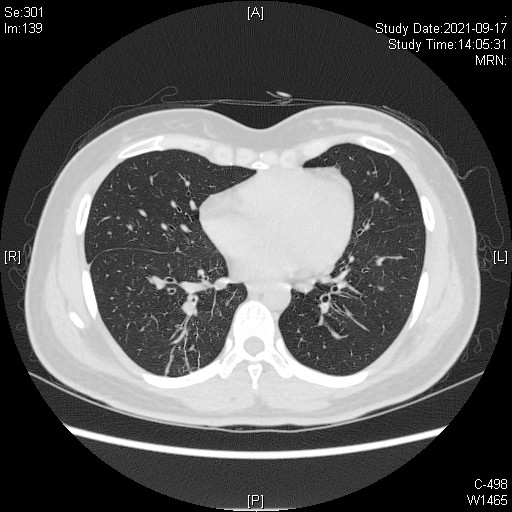

Supplement: Supplementary file 2 — Supplementary Information 2. [file 41598_2023_50702_MOESM2_ESM.zip › image data/MPP/wang/2021091703.jpg]

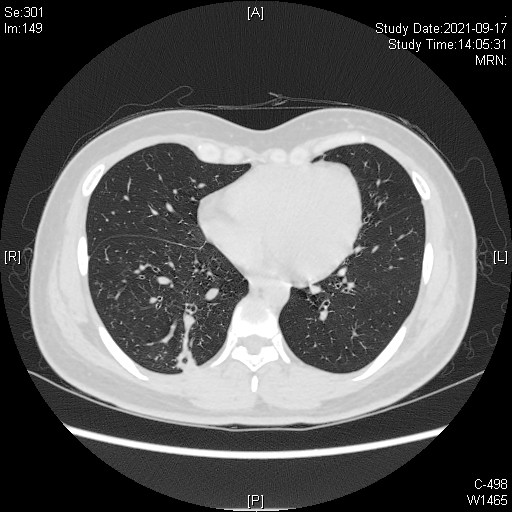

Supplement: Supplementary file 2 — Supplementary Information 2. [file 41598_2023_50702_MOESM2_ESM.zip › image data/MPP/wang/2021091704.jpg]

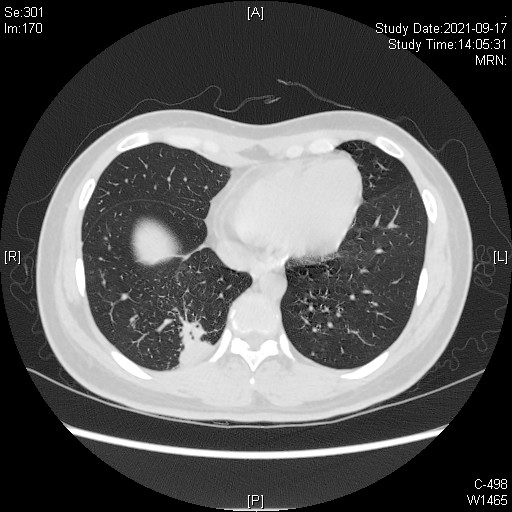

Supplement: Supplementary file 2 — Supplementary Information 2. [file 41598_2023_50702_MOESM2_ESM.zip › image data/MPP/wang/2021091705.jpg]

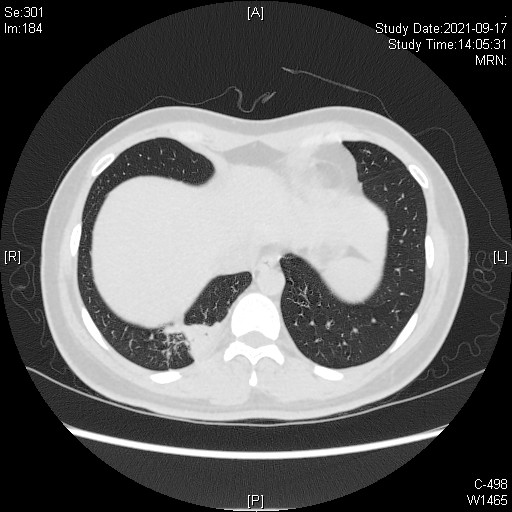

Supplement: Supplementary file 2 — Supplementary Information 2. [file 41598_2023_50702_MOESM2_ESM.zip › image data/MPP/wang/2021091706.jpg]

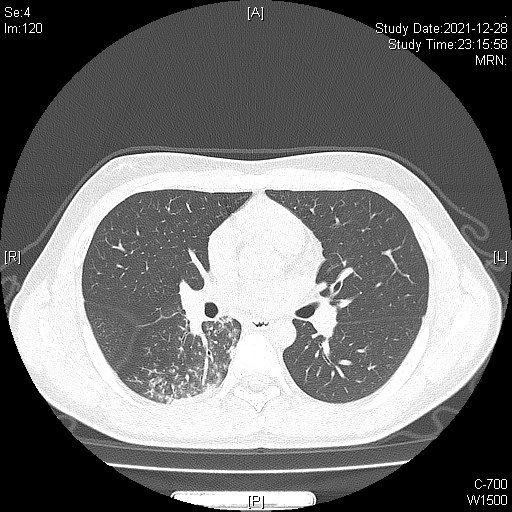

Supplement: Supplementary file 2 — Supplementary Information 2. [file 41598_2023_50702_MOESM2_ESM.zip › image data/MPP/wang2/wang (1).jpg]

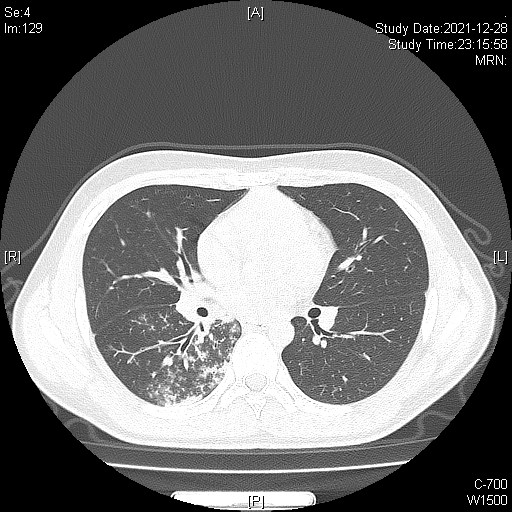

Supplement: Supplementary file 2 — Supplementary Information 2. [file 41598_2023_50702_MOESM2_ESM.zip › image data/MPP/wang2/wang (2).jpg]

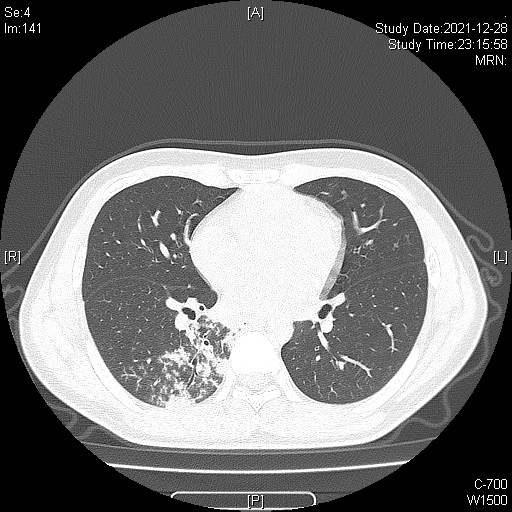

Supplement: Supplementary file 2 — Supplementary Information 2. [file 41598_2023_50702_MOESM2_ESM.zip › image data/MPP/wang2/wang (3).jpg]

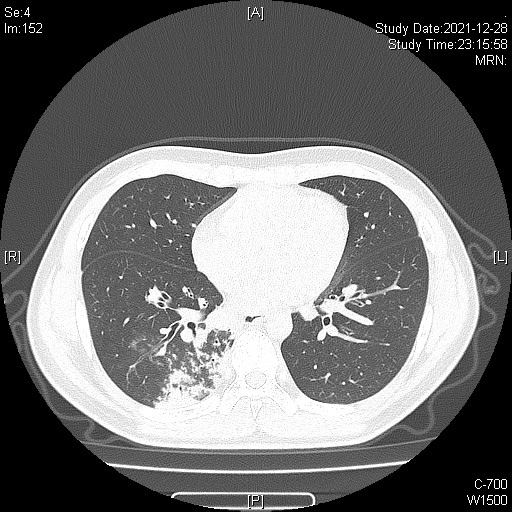

Supplement: Supplementary file 2 — Supplementary Information 2. [file 41598_2023_50702_MOESM2_ESM.zip › image data/MPP/wang2/wang (4).jpg]

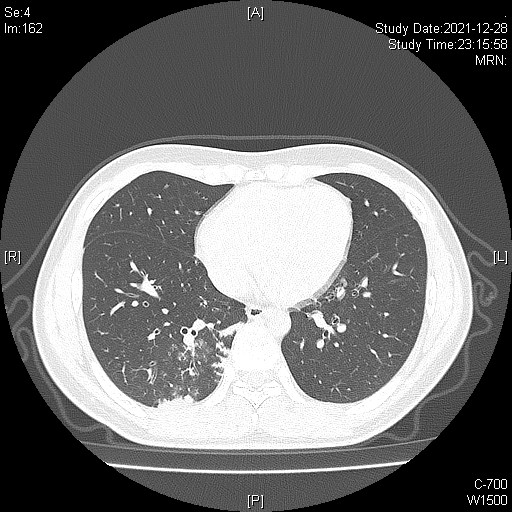

Supplement: Supplementary file 2 — Supplementary Information 2. [file 41598_2023_50702_MOESM2_ESM.zip › image data/MPP/wang2/wang (5).jpg]

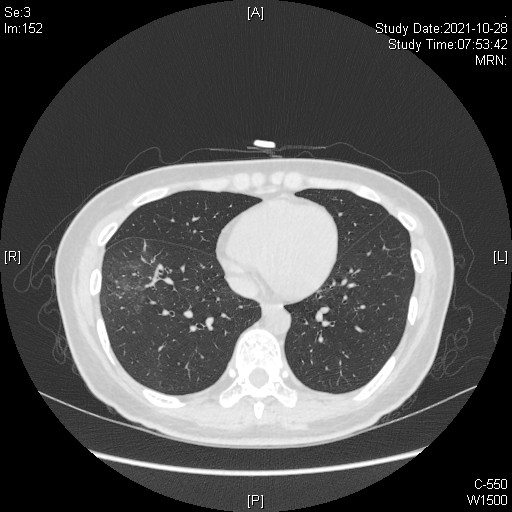

Supplement: Supplementary file 2 — Supplementary Information 2. [file 41598_2023_50702_MOESM2_ESM.zip › image data/MPP/yang/yang (1).jpg]

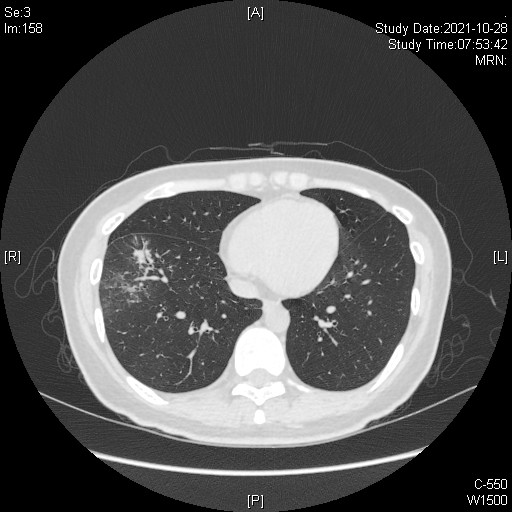

Supplement: Supplementary file 2 — Supplementary Information 2. [file 41598_2023_50702_MOESM2_ESM.zip › image data/MPP/yang/yang (2).jpg]

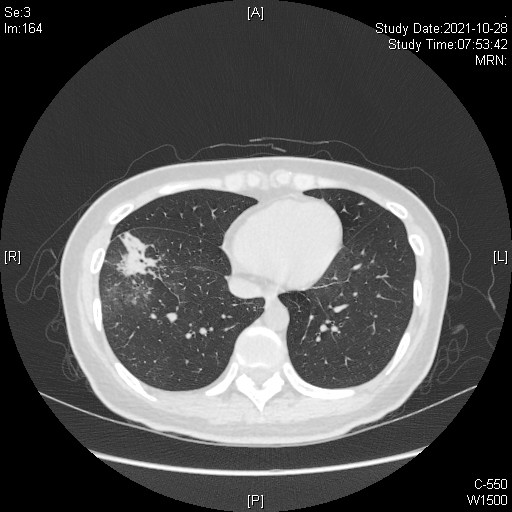

Supplement: Supplementary file 2 — Supplementary Information 2. [file 41598_2023_50702_MOESM2_ESM.zip › image data/MPP/yang/yang (3).jpg]

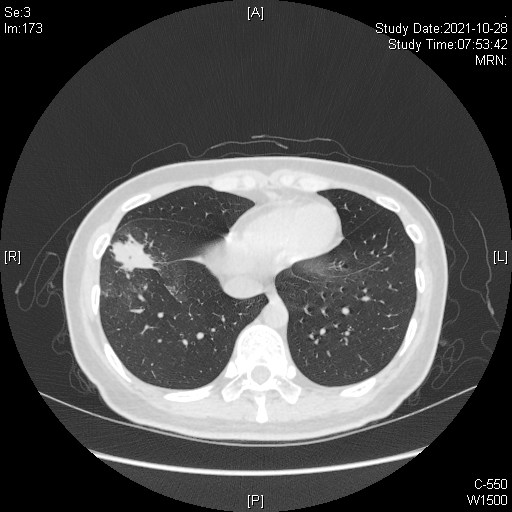

Supplement: Supplementary file 2 — Supplementary Information 2. [file 41598_2023_50702_MOESM2_ESM.zip › image data/MPP/yang/yang (4).jpg]

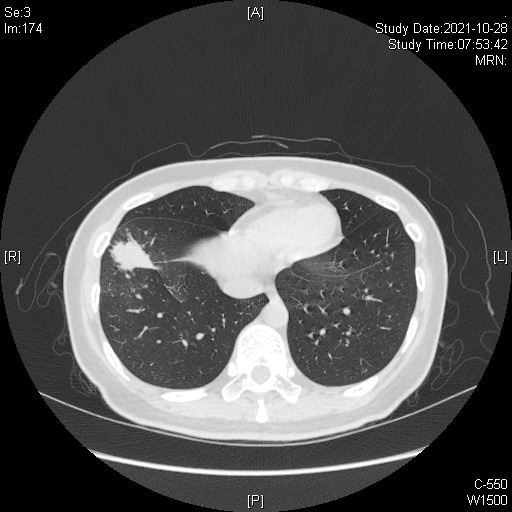

Supplement: Supplementary file 2 — Supplementary Information 2. [file 41598_2023_50702_MOESM2_ESM.zip › image data/MPP/yang/yang (5).jpg]

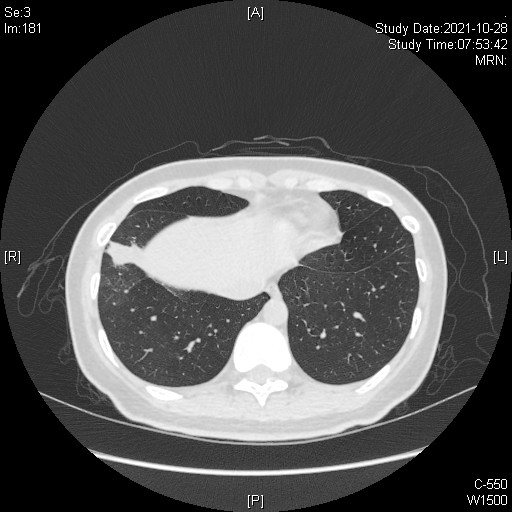

Supplement: Supplementary file 2 — Supplementary Information 2. [file 41598_2023_50702_MOESM2_ESM.zip › image data/MPP/yang/yang (6).jpg]

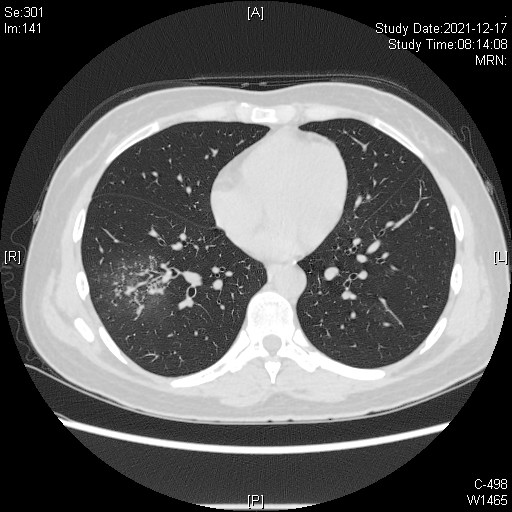

Supplement: Supplementary file 2 — Supplementary Information 2. [file 41598_2023_50702_MOESM2_ESM.zip › image data/MPP/yue/yue (1).jpg]

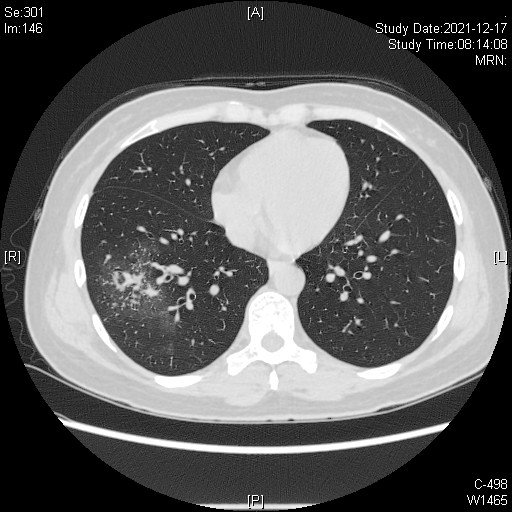

Supplement: Supplementary file 2 — Supplementary Information 2. [file 41598_2023_50702_MOESM2_ESM.zip › image data/MPP/yue/yue (2).jpg]

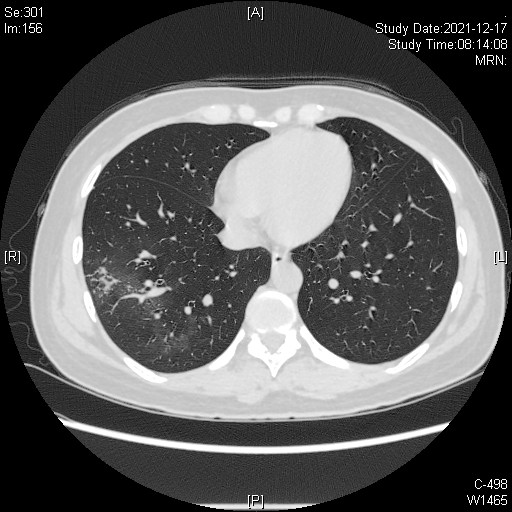

Supplement: Supplementary file 2 — Supplementary Information 2. [file 41598_2023_50702_MOESM2_ESM.zip › image data/MPP/yue/yue (3).jpg]

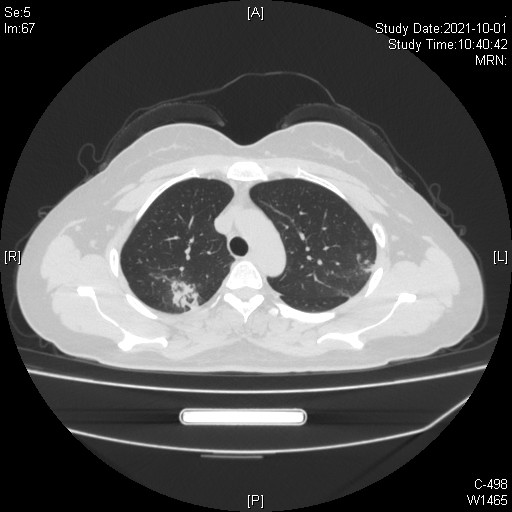

Supplement: Supplementary file 2 — Supplementary Information 2. [file 41598_2023_50702_MOESM2_ESM.zip › image data/MPP/zhang/zhang (1).jpg]

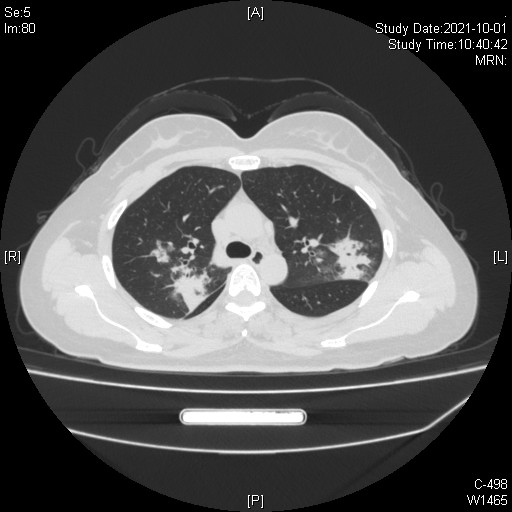

Supplement: Supplementary file 2 — Supplementary Information 2. [file 41598_2023_50702_MOESM2_ESM.zip › image data/MPP/zhang/zhang (10).jpg]

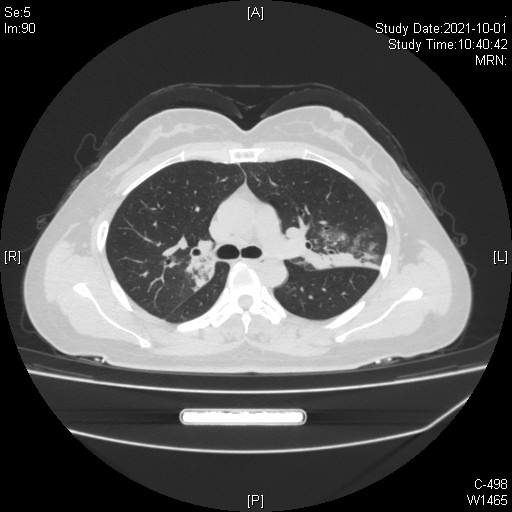

Supplement: Supplementary file 2 — Supplementary Information 2. [file 41598_2023_50702_MOESM2_ESM.zip › image data/MPP/zhang/zhang (11).jpg]

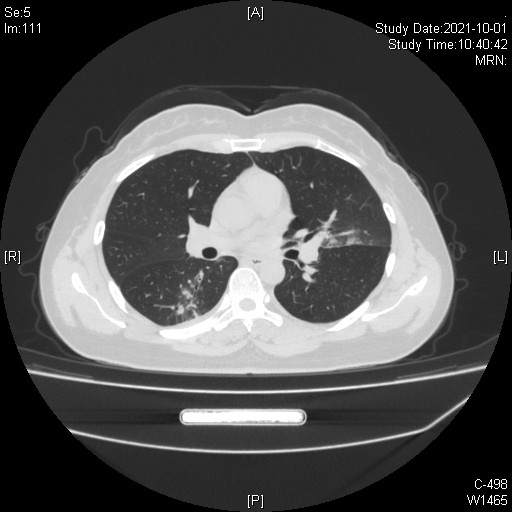

Supplement: Supplementary file 2 — Supplementary Information 2. [file 41598_2023_50702_MOESM2_ESM.zip › image data/MPP/zhang/zhang (12).jpg]

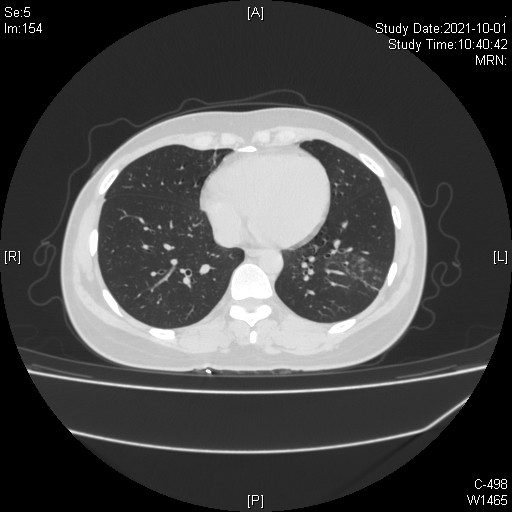

Supplement: Supplementary file 2 — Supplementary Information 2. [file 41598_2023_50702_MOESM2_ESM.zip › image data/MPP/zhang/zhang (13).jpg]

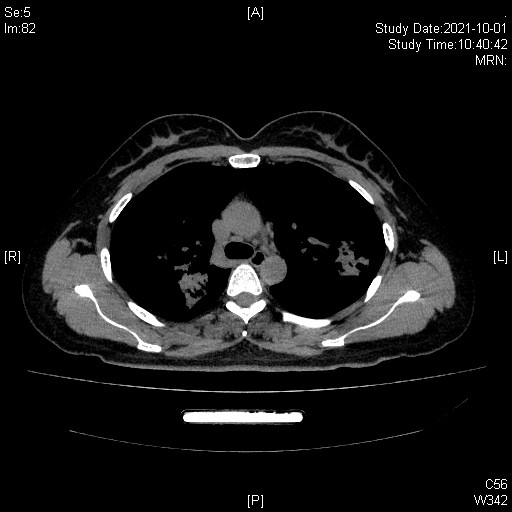

Supplement: Supplementary file 2 — Supplementary Information 2. [file 41598_2023_50702_MOESM2_ESM.zip › image data/MPP/zhang/zhang (14).jpg]

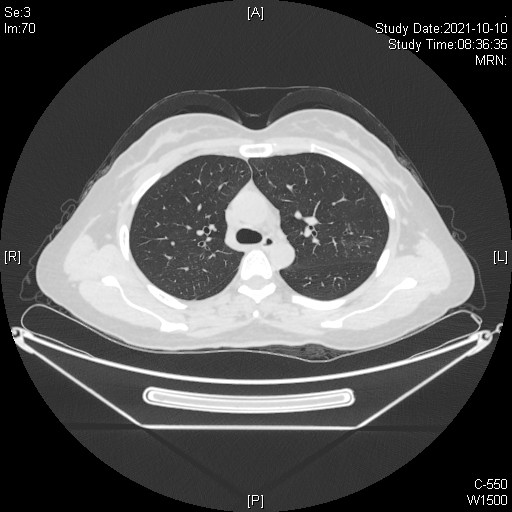

Supplement: Supplementary file 2 — Supplementary Information 2. [file 41598_2023_50702_MOESM2_ESM.zip › image data/MPP/zhang/zhang (15).jpg]

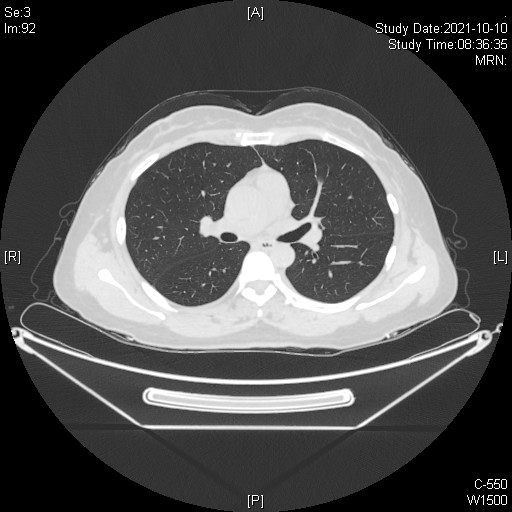

Supplement: Supplementary file 2 — Supplementary Information 2. [file 41598_2023_50702_MOESM2_ESM.zip › image data/MPP/zhang/zhang (16).jpg]

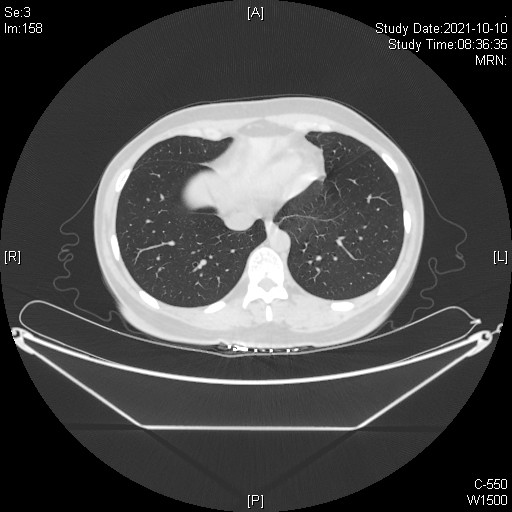

Supplement: Supplementary file 2 — Supplementary Information 2. [file 41598_2023_50702_MOESM2_ESM.zip › image data/MPP/zhang/zhang (17).jpg]

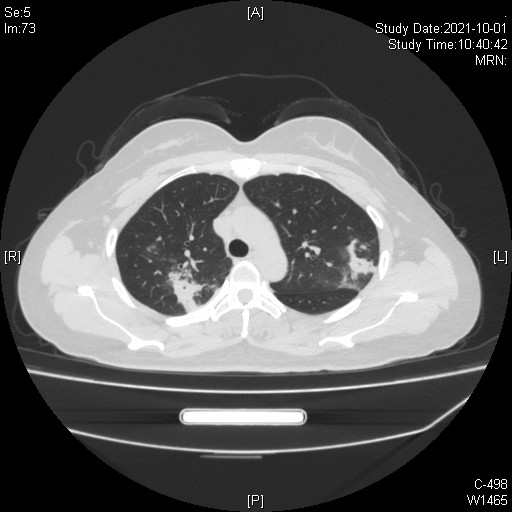

Supplement: Supplementary file 2 — Supplementary Information 2. [file 41598_2023_50702_MOESM2_ESM.zip › image data/MPP/zhang/zhang (2).jpg]

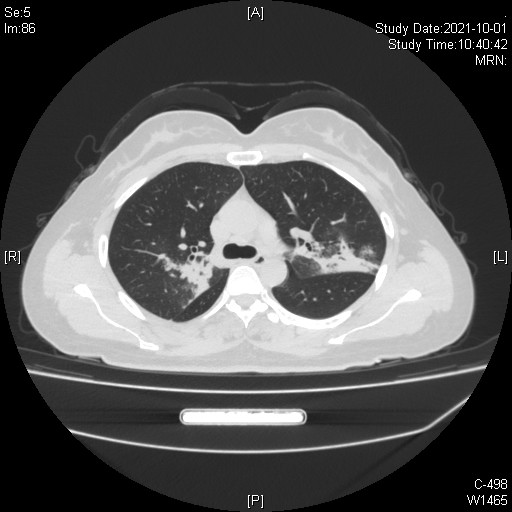

Supplement: Supplementary file 2 — Supplementary Information 2. [file 41598_2023_50702_MOESM2_ESM.zip › image data/MPP/zhang/zhang (4).jpg]

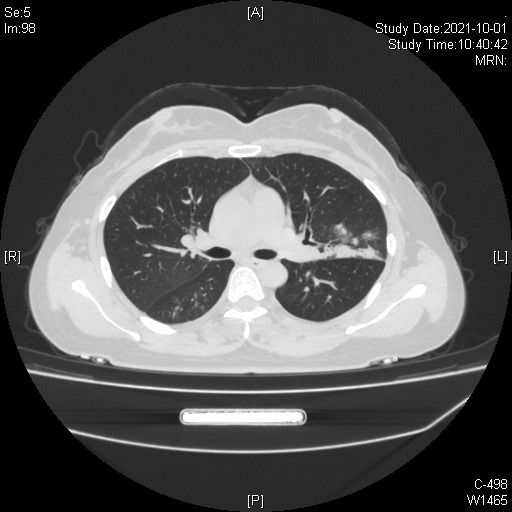

Supplement: Supplementary file 2 — Supplementary Information 2. [file 41598_2023_50702_MOESM2_ESM.zip › image data/MPP/zhang/zhang (5).jpg]

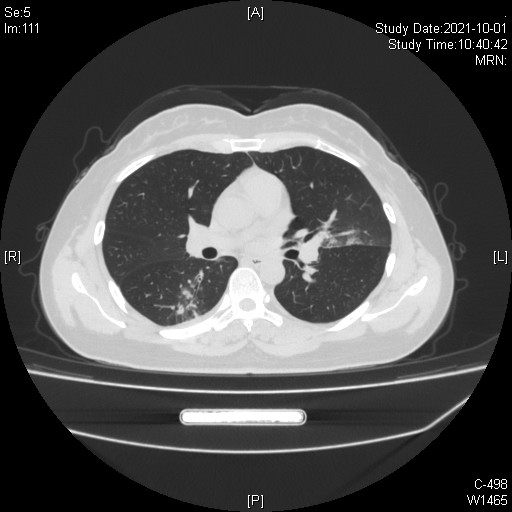

Supplement: Supplementary file 2 — Supplementary Information 2. [file 41598_2023_50702_MOESM2_ESM.zip › image data/MPP/zhang/zhang (6).jpg]

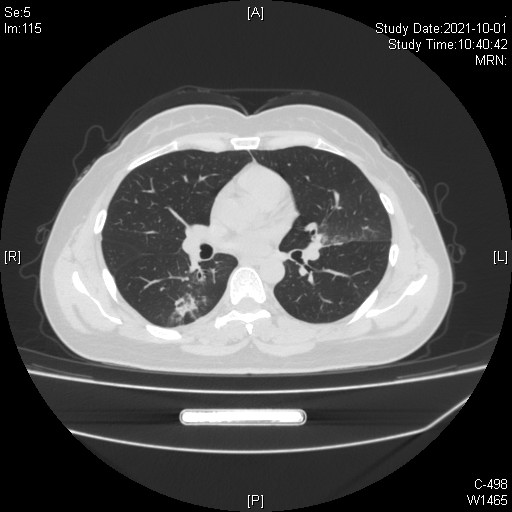

Supplement: Supplementary file 2 — Supplementary Information 2. [file 41598_2023_50702_MOESM2_ESM.zip › image data/MPP/zhang/zhang (7).jpg]

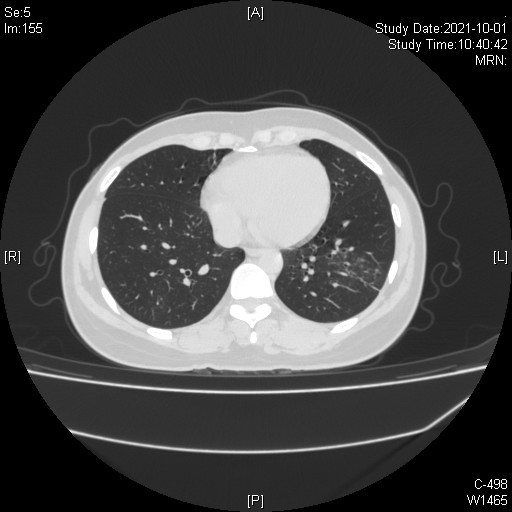

Supplement: Supplementary file 2 — Supplementary Information 2. [file 41598_2023_50702_MOESM2_ESM.zip › image data/MPP/zhang/zhang (8).jpg]

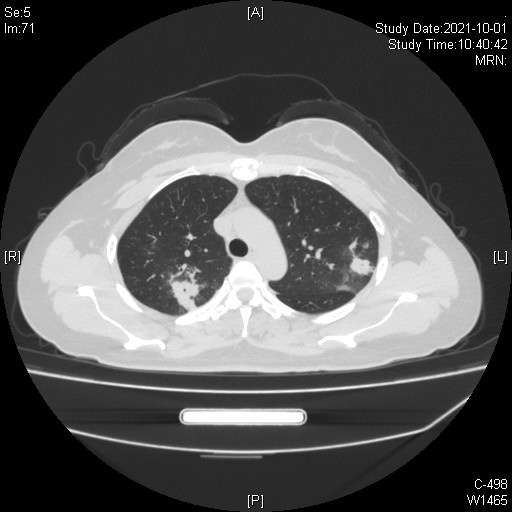

Supplement: Supplementary file 2 — Supplementary Information 2. [file 41598_2023_50702_MOESM2_ESM.zip › image data/MPP/zhang/zhang (9).jpg]

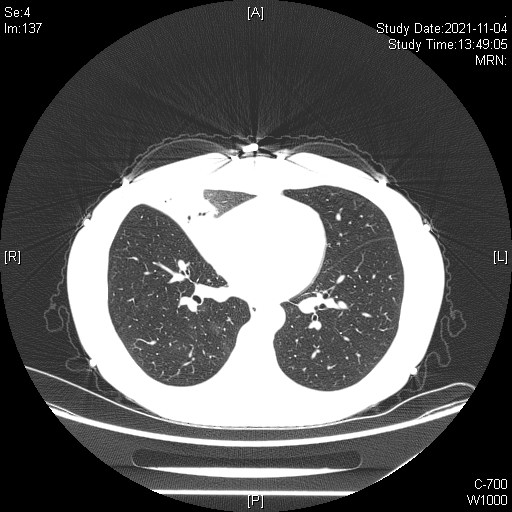

Supplement: Supplementary file 2 — Supplementary Information 2. [file 41598_2023_50702_MOESM2_ESM.zip › image data/MPP/zhang2/zhang (1).jpg]

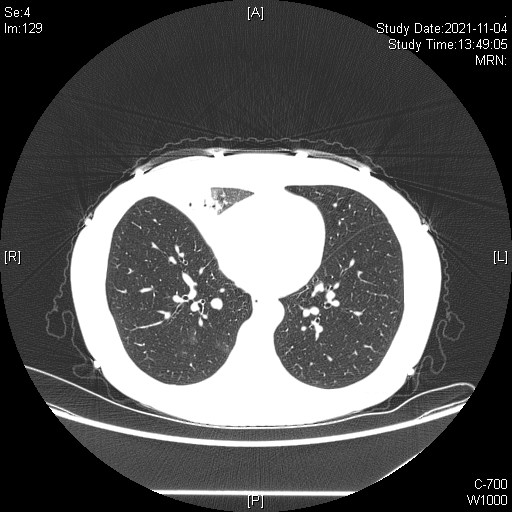

Supplement: Supplementary file 2 — Supplementary Information 2. [file 41598_2023_50702_MOESM2_ESM.zip › image data/MPP/zhang2/zhang (2).jpg]

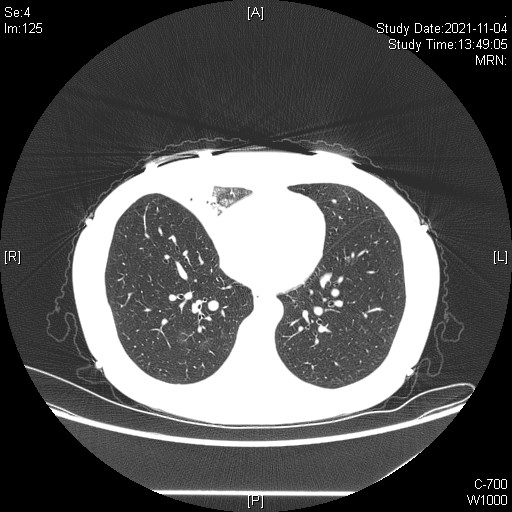

Supplement: Supplementary file 2 — Supplementary Information 2. [file 41598_2023_50702_MOESM2_ESM.zip › image data/MPP/zhang2/zhang (3).jpg]

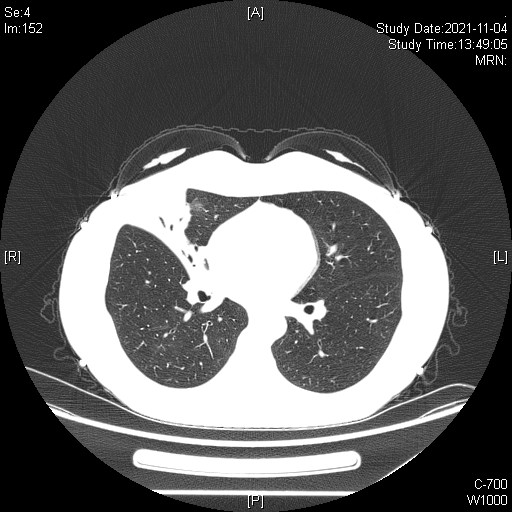

Supplement: Supplementary file 2 — Supplementary Information 2. [file 41598_2023_50702_MOESM2_ESM.zip › image data/MPP/zhang2/zhang (4).jpg]

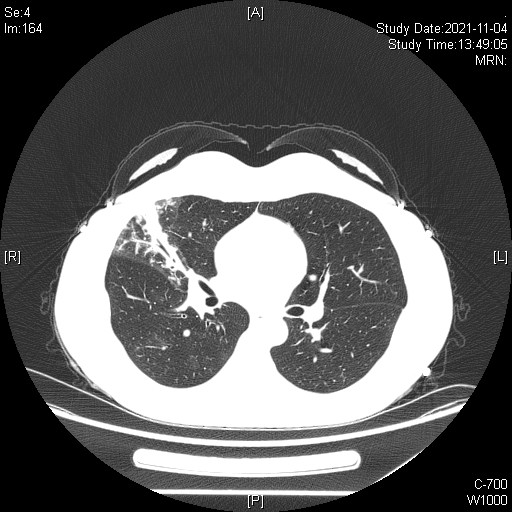

Supplement: Supplementary file 2 — Supplementary Information 2. [file 41598_2023_50702_MOESM2_ESM.zip › image data/MPP/zhang2/zhang (5).jpg]

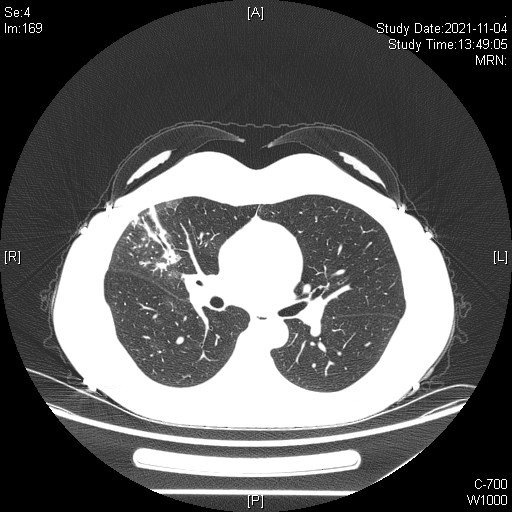

Supplement: Supplementary file 2 — Supplementary Information 2. [file 41598_2023_50702_MOESM2_ESM.zip › image data/MPP/zhang2/zhang (6).jpg]
